# Vascular Smooth Muscle Cells Transdifferentiate into Chondrocyte-Like Cells and Facilitate Meniscal Fibrocartilage Regeneration

**DOI:** 10.34133/research.0555

**Published:** 2024-12-23

**Authors:** Wenqiang Yan, Jin Cheng, Haoda Wu, Zeyuan Gao, Zong Li, Chenxi Cao, Qingyang Meng, Yue Wu, Shuang Ren, Fengyuan Zhao, Hongde Wang, Ping Liu, Jianquan Wang, Xiaoqing Hu, Yingfang Ao

**Affiliations:** ^1^ Department of Sports Medicine, Peking University Third Hospital, Institute of Sports Medicine of Peking University, Beijing, China.; ^2^ Beijing Key Laboratory of Sports Injuries, Beijing, China.; ^3^ Engineering Research Center of Sports Trauma Treatment Technology and Devices, Ministry of Education, Beijing, China.

## Abstract

The effective and translational strategy to regenerate knee meniscal fibrocartilage remained challenging. Herein, we first identified vascular smooth muscle cells (VSMCs) transdifferentiated into fibrochondrocytes and participated in spontaneous meniscal regeneration using smooth muscle cell lineage tracing transgenic mice meniscal defect model. Then, we identified low-intensity pulsed ultrasound (LIPUS) acoustic stimulus enhanced fibrochondrogenic transdifferentiation of VSMCs in vitro and in vivo. Mechanistically, LIPUS stimulus could up-regulate mechanosensitive ion channel Piezo1 expression and then activate the transforming growth factor β1 (TGFβ1) signal, following repression of the Notch signal, consequently enhancing fibrochondrogenic transdifferentiation of VSMCs. Finally, we demonstrated that the regular LIPUS stimulus enhanced anisotropic native-like meniscal fibrocartilage tissue regeneration in a beagle canine subtotal meniscectomy model at 6 months postoperatively. The single-cell RNA sequencing analysis confirmed the role of VSMC fibrochondrogenic transdifferentiation in meniscal regeneration.

## Introduction

The meniscus was located between femur and tibia cartilage, which provided the function of shock absorption, lubrication, and joint stability [1]. Meniscal defect or injury could disrupt the balance of joint mechanics. This would lead to secondary cartilage degeneration or even osteoarthritis, which affected knee joint motion and life quality [2]. Currently, the 3-dimensional (3D) printing technique was applied extensively in the field of regenerative medicine due to its advantage of precise manufacturing [3–5]. The studies implanting 3D-printed porous scaffold and stimulating factors to facilitate meniscal regeneration were increased dramatically, which obtained meniscus-like tissue and alleviated cartilage degeneration in some degree [6,7]. Within the regenerated meniscus-like tissue, there existed not only fibrocartilaginous and fibrous tissue but also abundant vascular tissue [8]. The vascularization could bring nutrients and oxygen and accelerate exchange of metabolites intrinsically, which was beneficial to meniscal regeneration. However, the role of cellular component of blood vessels in meniscal regeneration was not clarified to our knowledge. Previous studies have indicated that vascular smooth muscle cells (VSMCs) were not terminally differentiated and maintained phenotypic plasticity in response to various environmental conditions [9]. In atherosclerosis, VSMCs were demonstrated to transdifferentiate into chondrocyte-like cells and secrete cartilaginous matrix [10,11]. We hypothesized that VSMCs could transdifferentiate into chondrocyte-like cells and contribute to meniscal fibrocartilage regeneration. In the present study, we clarified VSMCs transdifferentiated into fibrochondrocytes (FCs) within the spontaneously regenerated meniscal tissue using mice meniscal defect model.

The biochemical stimulus was critical for inducing or maintaining fibrochondrogenic phenotype of regenerative cells during meniscal regeneration. Most of the studies regarding meniscal repair or regeneration applied exogenous growth factors, such as transforming growth factor β (TGFβ) family members, connective tissue growth factor (CTGF), and so on, or small molecules, such as kartogenin (KGN) [1,12]. However, the dosage, administration frequency, release model, metabolism, pharmacokinetics, and immunogenicity in vivo could not be defined, which affected its translation in clinic. Recent studies have attempted to apply biophysical stimulus as an alternate for biochemical stimulus, such as low-intensity pulsed ultrasound (LIPUS) [13]. LIPUS could provide pulsed acoustic mechanical stimulus and has been approved by the U.S. Food and Drug Administration (FDA) for the treatment of fresh bone fractures and non-unions [14]. LIPUS has been demonstrated to up-regulate the expression of genes related to fracture healing, including osteopontin and growth factors, which promoted chondrogenesis and angiogenesis [15]. The mechanotransductive effects exerted by LIPUS increased the expression of anabolic factors of chondrocytes, including aggrecan and type II collagen (COL II), and promoted chondrogenic differentiation of mesenchymal stem cells (MSCs) [16,17]. Moreover, Kamatsuki et al. [18] demonstrated that LIPUS stimulus enhanced the gene expression of SOX9, aggrecan, and COL II of native meniscus cells. Regarding its effect on chondrogenesis, we hypothesized that LIPUS could promote the chondrogenic transdifferentiation of VSMCs. In the present study, we clarified that LIPUS could up-regulate fibrochondrogenic markers and down-regulate smooth muscle contractile markers of VSMCs in vitro. Moreover, LIPUS stimulus promoted meniscal fibrocartilage regeneration in a subtotal meniscectomy model of beagle canine.

Single-cell RNA sequencing (scRNA-Seq) is a well-established and powerful way to study transcriptomic cell-to-cell variation, which could identify cell types and provide meaningful clues during tissue physiological, pathological, and regenerative processes [19]. In the present study, the scRNA-Seq was used to clarify the cell populations and gene expression during the regenerative process of meniscus. The pseudo-time trajectory analysis clarified that VSMCs existed at the pseudo-space trajectory start and transdifferentiated into FCs. The abbreviations used in the present study were summarized in Table S1.

## Materials and Methods

### The manufacturing of PU_PCL scaffold

In the present study, the composite PU_PCL material was used for scaffold manufacturing. A previous study demonstrated that a combination of thermoplastic medical-grade polyurethane (PU) (Carbothane PC-3575A, Lubrizol, USA) and poly-ε-caprolactone (PCL) (19561-500G, molecular weight 43,000 to 50,000; Polysciences Inc., USA), referred to as PU_PCL, at a weight ratio of PU/PCL = 60/40 resembled the native meniscal tissue in mechanical properties [20]. This would avoid secondary cartilage degeneration caused by pure PCL-based scaffold implant, which was stiffer than native meniscus and cartilage [21]. Herein, trichloromethane was used to dissolve PU and PCL particles. After stirring overnight in the fume hood, PU and PCL could be mixed thoroughly. In order to accelerate the evaporation of trichloromethane, the mixed solution was put in the drying oven at 60 °C for 2 d. Then, the PU_PCL block was cut into small pieces of 2 × 2 × 2 mm using scalpel. The PU_PCL composite material was used for scaffold printing.

The PU_PCL meniscal scaffold printing was completed according to a previous study [22]. Briefly, the 2D images of meniscus from mature beagle canine (age: 12 months; weight: 20 kg) were acquired by micro-computed tomography (CT) scanning (GE Healthcare, USA). The 3D model was constructed. In the present study, the nozzle of 400 μm was used for extrusion. The printing path within the meniscus model contains circumferential and radial orientations, resembling the native arrangement of collagen fibers in native meniscus. Then, the meniscus scaffold was fabricated in a layer-by-layer stacking manner.

### VSMC isolation and expansion

After institutional review board approval, VSMCs were harvested from aorta. The thoracic and abdominal aorta were harvested. The aorta was rinsed with cold sterile PBS solution to remove blood clot. The peripheral soft tissue was removed using scissors. The media vascular smooth muscle layer could be separated after removal of adventitia and intima using scalpel. Then, the media layer was cut into approximately 0.1-mm pieces and then digested with 0.2% type I collagenase (Gibco, USA) for 2 h at 37 °C. The single-cell suspension was prepared. After expansion and passage, the VSMCs at passage 2 (P2) were used for subsequent experiments.

### Flow cytometry

Preparation of cells: after cell processing and counting, centrifuge at 1,000 rpm for 5 min, discard the supernatant, and wash the cells once with cell wash solution [PBS containing 2% bovine serum albumin (BSA)]; cell fixation: fix cells with 4% paraformaldehyde at a ratio of 1 ml of 4% paraformaldehyde per 2 × 10^6^ cells, and fix at room temperature for 15 min; cell cleaning: add cell cleaning solution, centrifuge at 1,000 rpm for 5 min, discard the supernatant, and repeat the cleaning process; cell membrane breaking: resuspend cells with a membrane breaking agent (00-8333-56, eBioscience, Thermo Fisher Scientific Inc., USA) and add cells to a 1.5-ml Eppendorf tube, 1 × 10^6^ cells/tube, with a volume of 100 μl/tube; control settings: the blank tube and only secondary antibody incubation tube were included and regarded as control tubes, and the primary and secondary antibody incubation tube was regarded as test tube; primary antibody incubation: the primary antibody was added to the test tube. Mix thoroughly and incubate at room temperature for 30 min. Shake the reaction tubes every 10 min during incubation to allow the cells and antibodies to fully react. The PBS solution with same volume was added to the control tubes; cell cleaning: add an appropriate amount of transmembrane penetrating agent, centrifuge at 1,000 rpm for 5 min, discard the supernatant, and repeat washing cells twice; secondary antibody incubation: add an appropriate amount of fluorescence-labeled secondary antibodies to the test tube and only secondary antibody incubation control tube, mix well, and incubate at room temperature in the dark for 30 min. Shake the reaction tube every 10 min during incubation. The PBS solution with the same volume was added to the blank tube; cell cleaning: add an appropriate amount of transmembrane penetrating agent, centrifuge at 1,000 rpm for 5 min, discard the supernatant, and repeat washing cells twice; flow cytometry analysis: resuspend cells with 100 μl of PBS solution and first analyze the control tubes and then the test tube. In the present study, the smooth muscle 22α (SM22α) marker was used to analyze the purity of isolated rat VSMCs. The secondary antibody was conjugated with Alexa Fluor 647 fluorescence (excitation: 652 nm, emission: 668 nm).

### Cell treatment

To evaluate the effect of LIPUS stimulus on rat VSMC phenotypes in vitro, the ultrasonic therapy instrument was used (UT1021, Nu-Tek, China). The LIPUS waves with 0.1 W/cm^2^ intensity were transmitted through the bottom of 6-well culture dish with coupling agent. The rat VSMCs were stimulated at on–off ratio of 20% and frequency of 3 MHz for 10 min once a day for 14 d. The nonstimulated rat VSMCs seeded on the same culture plate were used as controls. During the whole treatment period, the rat VSMCs were cultured with serum-free and TGFβ1-free culture medium (α-minimum essential medium). The protein and mRNA were collected at 14 d.

The rat VSMCs were treated with YODA1 (50 μM, HY-18723, MCE) for 24 h to activate Piezo1. The rat VSMCs were treated with GsMTx-4 (2 μM, HY-P1410, MCE) for 24 h to suppress Piezo1. To identify the role of TGFβ1 in chondrogenic transdifferentiation of rat VSMCs in vitro, the rat VSMCs were cultured with chondrogenic induction medium for 2 weeks containing growth medium supplemented with TGFβ1 (10 ng/ml, 100-21, PeproTech, USA), dexamethasone (10 nM, Sigma-Aldrich, USA), ascorbate-2-phosphate (50 μg/ml, Sigma-Aldrich, USA), and insulin–transferrin–selenium (6.25 μg/ml, ITS, Gibco, USA). To identify whether GsMTx-4 treatment could block the effects of LIPUS on rat VSMC transdifferentiation in vitro, the control group, the LIPUS treatment group, and the LIPUS treatment + GsMTx-4 (2 μM, HY-P1410, MCE) group were involved. The LIPUS treatment parameters, time, and period were identical to the aforementioned. The culture medium in 3 groups was serum and cytokine free.

### Mice model

All animal studies were approved by Peking University Ethics Committee. All applicable institutional and/or national guidelines for the care and use of animals were followed. In the present study, the *Myh11-CreER^T2^; Rosa26-LSL-Tdtomato* transgenic mice were used to trace VSMCs. The Myh1l-positive cells expressed tdtomato protein. Four transgenic mice (male, 2 months) were included. A standard dose of 100 μl of tamoxifen/corn oil solution (20 mg/ml) was administered by intraperitoneal injection once a day for a total of 5 consecutive days. The total meniscectomy of medial meniscus was performed in bilateral knees at 11 d from the initiation of tamoxifen induction. Knee joints were harvested after 4 weeks.

### Rabbit model

First, to investigate the role of VSMCs in meniscus repair of rabbit, focal meniscus defect was prepared in the medial meniscus. Four adult rabbits (female, 6 months) were included. The full-thickness meniscal defects of 2.0 mm diameter were prepared according to the previous study [23]. The samples were harvested at 12 weeks after surgery for subsequent histological analysis.

Second, to study the effect of VSMC transplantation on facilitating meniscal repair in rabbit, the allogenic VSMC transplantation was performed. Four rabbits (8 knees) were used as the VSMC transplantation group. The VSMCs of 1 × 10^5^ were encapsulated with gel-MA (5%) and then injected into the defect site. The blue light was used to cure gel-MA. In the present study, to trace the fate of transplanted cells, VSMCs from male rabbits (6 months) were transplanted into female rabbits. The male VSMCs contained male-specific SRY gene and could be distinguished from intrinsic female cells using in situ hybridization (ISH). Another 4 rabbits (8 knees) were used as the blank group. Only gel-MA hydrogel was implanted to meniscal defect. All samples were harvested at 12 weeks after surgery. Meniscal repair scoring system [24] was used to evaluate meniscal healing degree. The repair scoring system was described in Table S2.

Third, to evaluate the role of LIPUS in ectopic chondrogenic transdifferentiation of VSMCs in vivo, male rabbit VSMCs were seeded in semilunar porous scaffold and then transplanted subcutaneously in the back of female rabbits. Identically, the transplanted male VSMCs could be distinguished from host cells by ISH. Nine female rabbits (6 months) were used. The blank scaffold, scaffold + VSMCs + TGFβ1, and scaffold + VSMCs + TGFβ1 + LIPUS groups were included. Each group contained 3 rabbits (6 samples). For the blank scaffold group, only the porous PU_PCL scaffold was implanted subcutaneously in the back. For the scaffold + VSMCs + TGFβ1 group, the porous scaffold was first placed in the matched mold, then a total of 1 × 10^6^ VSMCs encapsulated with 5% gel-MA [containing 1% phenyl (2,4,6-trimethylbenzoyl) phosphinic acid lithium salt (LAP) photoinitiator and 10 ng/ml TGFβ1] solution were injected into the porous scaffold followed by crosslinking using blue light irradiation for 20 s. The porous scaffold containing VSMCs was implanted subcutaneously in the back. For the scaffold + VSMCs + TGFβ1 + LIPUS group, on the basis of the scaffold + VSMCs + TGFβ1 group, LIPUS stimulus (frequency of 3 MHz, intensity of 0.1 W/cm^2^, on–off ratio of 20%) was performed for 10 min once every 2 d. All samples were harvested for histological analysis at 12 weeks after surgery.

### Rat model

To evaluate the effect of LIPUS stimulus on facilitating VSMC migration, the porous PU_PCL scaffold was implanted subcutaneously in the back of rat. Six rats (male, 2 months) were used. The LIPUS stimulus group and the non-LIPUS blank group were included. Each group contained 3 rats (6 samples). Two 3D-printed semilunar porous PU_PCL scaffolds were implanted subcutaneously in the back of each rat. In the LIPUS stimulus group, LIPUS (frequency of 3 MHz, intensity of 0.1 W/cm^2^, on–off ratio of 20%) was performed for 10 min once every 2 d. All samples were harvested at 12 weeks after surgery. The immunostaining for SM22α was used to identify VSMCs.

### Beagle canine model

The present study included 16 adult beagle canines (male, 1 year, 20 ± 5 kg). The detailed arrangement for each sample was described in Table S3. The meniscectomy (blank), PU_PCL scaffold (scaffold), PU_PCL scaffold supplemented LIPUS stimulus (scaffold + LIPUS), and sham groups were involved. The standard anesthesia, skin preparation, disinfection, and surgical approach were prepared according to previous studies [25,26]. The subtotal meniscectomy model of medial meniscus was completed according to a previous study [22]. In the scaffold + LIPUS group, the porous meniscal scaffold was transplanted anatomically and fixed with the residual meniscal tissue using 2-0 Ethibond sutures. The incision was closed layer-by-layer using sutures. The LIPUS stimulus (frequency of 3 MHz, intensity of 0.1 W/cm^2^, on–off ratio of 20%) was performed in the medial side of knee for 10 min once every 2 d. In the meniscectomy (blank) group, only subtotal meniscectomy was performed. In the PU_PCL scaffold (scaffold) group, the porous meniscal scaffold was transplanted anatomically after subtotal meniscectomy in medial meniscus. The meniscus remained intact in the sham group, while the remaining procedures were identical to other groups. After operation, beagle canines were first limited in small cages (1.2 × 1.2 × 1.2 m) to avoid excessive motion. The beagle canines were administered with nonsteroidal anti-inflammatory drugs and penicillin for 2 weeks. Then, the beagle canines were permitted to move freely. The samples were collected after 6 months. Histology and immunostaining were used to evaluate the chemical components of the regenerated meniscal tissue. The mechanical properties of regenerated tissue were evaluated using nanoindentation test. scRNA-Seq was used to deeply study the repair process. The osteoarthritis cartilage histopathology assessment system [osteoarthritis cartilage histopathology assessment system (OARSI)] scoring [27] was used to evaluate cartilage status of medial femoral condyle (MFC) and medial tibial plateau (MTP).

### Costaining of SOX9 immunofluorescence and biotin-labeled ISH for SRY gene

The probe for SRY gene was designed according to a previous study [23]. The probe sequence was as follows: SRY probe (5′ and 3′ biotin) TGCAAGCAGCAAACTGTCGCT (Qiagen, Germany). All solutions used for ISH were free of ribonuclease. The paraffin-embedded sections were immersed into xylene and ethanol to deparaffinize and regain water. The sections were permeabilized by 20 μg/ml proteinase K solution at 37 °C for 15 min and then washed with PBS. The heat-induced antigen retrieval was completed using pH 6.0 citric acid for 20 min. Then, the slices were treated with prehybridization buffer for 1 h at 37 °C. The tissue section was incubated with hybridization mix (hybridization buffer with SRY probe, 80 nM) for 1 h at 54 °C. After hybridization, rigorous wash was completed with graded sodium citrate buffer (SSC) and PBS. Nonspecific protein binding was blocked by 1% BSA at room temperature for 15 min. Then, the slices were incubated with fluorescein isothiocyanate (FITC)-labeled anti-biotin and SOX9 antibody for 1 h at room temperature. Then, the slices were incubated with secondary antibody and 4′,6-diamidino-2-phenylindole (DAPI) for 30 min. After thorough washing with phosphate-buffered saline with Tween 20 (PBST), the slices were sealed with anti-fluorescence quenching agent. The confocal microscope (TCS-SP8, Leica) was used to capture the images.

### Nanoindentation test

The nanoindentation test was completed according to a previous study [25]. First, the sample was glued to a glass slide with surface perpendicular to the indentation direction. The rubber ring was then glued to the glass slide to enclose the sample tissue. The PBS solution was added to the rubber ring to keep the samples hydrated. The samples should be avoided to be drying or dehydrated in the entire process. A Tribo-Indenter (Hysitron) with a 400-μm-radius curvature, conospherical, diamond probe tip was used. Random 6 points through each sample were measured. The trapezoidal load function with load (10 s), hold (2 s), and unload (10 s) was performed. The displacement control model with maximal 500-nm depth was used. Finally, the elastic modulus and hardness were calculated.

### RNA sequencing of rat VSMCs

The RNA-sequencing analyses of rat VSMCs after LIPUS treatment were performed using the Dr.TOM Platform (https://biosys.bgi.com). Total RNA was extracted using TRIzol reagent. cDNA libraries were constructed for each pooled RNA sample using MGIEasy RNA Directional kit Total RNA-seq. Bowtie2 (v2.2.5) was used to align the clean reads to the gene set. The gene expression level was calculated by RSEM (v1.3.1). The gene expression was determined by the TPM (transcripts per million reads) method. The differentially expressed genes were identified by DESeq2 (v1.4.5) algorithm. Significant analysis was completed using the *P* value and false discovery rate (FDR) analyses. The genes with fold change > 2 or fold change < 0.5, FDR < 0.05 were considered to be differentially expressed. To take insight to the change of phenotype, Gene Ontology (GO) (http://www.geneontology.org/) and Kyoto Encyclopedia of Genes and Genomes (KEGG) (https://www.kegg.jp/) enrichment analysis of annotated different expression gene was performed by Phyper (https://en.wikipedia.org/wiki/Hypergeometric_distribution) based on hypergeometric test. The significantly affected GO categories and pathways were identified by Fisher’s exact test. The *P* value was used to define the threshold of significance.

### scRNA-Seq of regenerated and native beagle canine meniscal tissue

The enzyme solution containing 2% type I collagenase and 2% type II collagenase was prepared using centrifuge tubes and preheated at 37 °C in water bath. The meniscal tissue was cut into small pieces (1 × 1 × 1 mm) using scalpel. The tissue block was transferred into a centrifuge tube containing enzymatic hydrolysate and placed in the constant temperature shaker at 37 °C for digestion. The enzymatic solution was shaken gently. The tissue digestion was examined via trypan blue staining until no obvious tissue block existed, and then the digestion could be paused. The cell suspension was filtered using 40-μm cell strainer. The residual cells were cleaned using 2 to 3 ml of precooled complete medium, in which the cleaning solution was filtered and collected in a centrifuge tube. The cell suspension was centrifuged at 300*g* and 4 °C for 5 min. The supernatant was discarded. Cell status was observed via trypan blue staining. The dead cells and debris were removed using Dead Cell Removal Kit. PBS–BSA (0.04%) was used to wash cells. Centrifuge at 300*g* and 4 °C for 5 min and discard the supernatant. An appropriate amount of PBS–BSA (0.04%) was used to resuspend the precipitation followed by counting the cells using a hemacytometer.

The single-cell suspension, oil, and beads were added into C4 scRNA slide and instrument in order. Cell lysis and magnetic bead capture mRNA were performed in droplets. All reagents were prepared in advance for emulsion breakage. The vacuum pump was used for emulsion breakage. The reaction system was configured. After reacting at the suitable temperature for a fixed period of time, reverse transcription was conducted. Then, cDNA second strand was synthesized after reacting at the suitable temperature for a fixed period of time. After reacting at the suitable temperature for a fixed period of time, cDNA and oligo products were amplified. The purification process was performed separately. The cDNA and oligo products were subjected to quality detection of their concentration and the size distribution of the fragments. Afterward, oligo products were subjected to amplification, index ligation, and purification for further circularization. The cDNAs were subjected to fragmentation, end repair, and addition of “A” base at the 3’-end of each strand followed by purification. The cDNAs were subjected to adaptor ligation followed by purification. The PCR system was configured. After reacting at the suitable temperature for a fixed period of time, amplification was processed via PCR followed by purification. The corresponding library quality control was completed. cDNA and oligo products were respectively denatured into single strand. The reaction system and program for circularization were respectively configured and set up. Single-stranded cyclized products were produced, while uncyclized linear DNA molecules were digested. Single-stranded circle DNA molecules were replicated via rolling cycle amplification, and a DNA nanoball (DNB) that contained multiple copies of DNA was generated. Sufficient quality DNBs were then loaded into patterned nanoarrays using high-intensity DNA nanochip technique and sequenced through combinatorial probe-anchor synthesis (cPAS).

The raw gene expression matrix was generated by processing raw sequencing data for each sample using DNBelab_C4scRNA (v1.0.1) [28]. Downstream analysis was completed using the R package Seurat (v 3.2.0) [29]. The quality control was performed based on the number of detected genes and proportion of mitochondrial reads per cell. Specifically, cells with less than 200 detected genes or cells with >90% of the maximum genes were filtered out. For the mitochondrial metric, cells were sorted in descending order according to the mitochondrial read ratio. The top 15% of cells were filtered out. Potential doublets were identified and removed by DoubletDetection (https://rdrr.io/github/scfurl/m3addon/man/doubletdetection.html). The cell cycle analysis was performed using the CellCycleScoring function in Seurat program. The gene expression dataset was normalized followed by principal components analysis (*n* = 15) using the 2,000 highly variable genes in the dataset. Uniform Manifold Approximation and Projection (UMAP) was then used for 2D visualization of the resulting clusters. For each cluster, the marker genes were identified using the FindAllMarkers function as implemented in the Seurat package (logfc. threshold > 0.25, minPct > 0.1, and *P*_adj_ ≤ 0.05). Then, clusters were remarked to known cell types by the single-cell sequence atlas (SCSA) method [30]. Differentially expressed genes across different samples were identified using the FindMarkers function in Seurat with parameters “logfc. threshold > 0.25, minPct > 0.1, and *P*_adj_ ≤ 0.05”.

GO (associated 3 integrated databases: UniProt: http://ftp.ebi.ac.uk/pub/databases/GO/goa/UNIPROT/goa_uniprot_all.gaf.gz, National Center for Biotechnology Information’s (NCBI) gene2GO: ftp://ftp.ncbi.nih.gov/gene/DATA/gene2go.gz, GO’s official website: ftp://ftp.pir.georgetown.edu/databases/idmapping/idmapping.tb.gz) analysis and KEGG (V93.0) pathway analysis were performed using phyper, a function of R. Only GO terms or KEGG pathways with FDR ≤ 0.05 were considered to be significantly enriched. Monocle2 [31] applied reversed graph embedding to describe multiple fate decisions in a fully unsupervised manner. We used Monocle2 to do pseudo-time analysis.

### Statistical analysis

The data were demonstrated with mean values ± SD. The Shapiro–Wilk test was used to evaluate data distribution. The equal variance of data was checked before analysis. The unpaired 2-tailed Student’s *t* test was performed for the comparison of 2 groups. The ordinary one-way analysis of variance (ANOVA) or 2-way ANOVA with Bonferroni multiple comparison test was performed for the comparison of multiple groups. The statistical significance was considered when *P* < 0.05. The statistical analysis was performed using GraphPad Prism software (version 8.0.1, USA). The reagents or resources in the present study were described in Table S4.

## Results

### VSMCs transdifferentiated into chondrocyte-like cells during meniscal regeneration in mice and rabbit

**Fig. 1.**
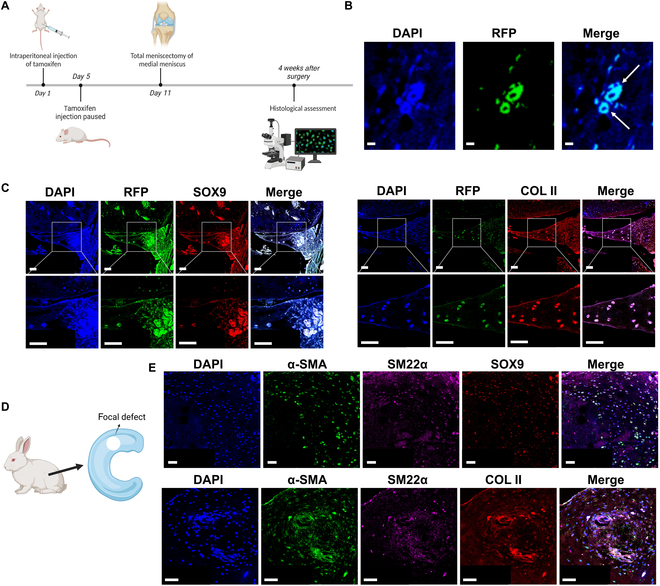
Vascular smooth muscle cells (VSMCs) transdifferentiate into chondrocyte-like cells during spontaneous meniscal regeneration in mice and rabbit meniscus defect model. (A) Time axis of operations in *Myh11-CreER^T2^; Rosa26-LSL-Tdtomato* smooth muscle lineage tracing transgenic mice. Four transgenic mice (male, 2 months) were included. The total meniscectomy of medial meniscus was performed in bilateral knees at 11 d from the initiation of tamoxifen induction. The knee joints were harvested at 4 weeks after surgery. (B) Validation of using *Myh11-CreER^T2^; Rosa26-LSL-Tdtomato* transgenic mice to trace smooth muscle cell lineage. In transgenic mice tail tissue, the RFP signals are restricted in the blood vessels. The white arrows indicate blood vessels. Scale bar, 50 μm. (C) Immunofluorescent costaining of RFP/SOX9 (left) and RFP/COL II (right) in transgenic mice spontaneously healed meniscal tissue after total meniscectomy. Scale bar, 50 μm. (D) Schematic diagram of meniscal focal defect preparation in rabbit. Four adult rabbits (female, 6 months) were included. The full-thickness defects of 2.0 mm diameter were prepared in the medial menisci of bilateral knees. The samples were harvested at 12 weeks after surgery. (E) Immunofluorescent costaining of α-SMA/SM22α/SOX9 (top) and α-SMA/SM22α/COL II (bottom) in rabbit spontaneously healed meniscal tissue. Scale bar, 50 μm.

Small-animal models, like mice or rabbit, had relatively robust intrinsic healing capacity compared to large animals and human beings. Thereby, small animals would be appropriate to investigate the role of VSMCs during the meniscal regenerative process. Recently, myosin heavy chain 11 (Myh11) has been identified as a specific marker of smooth muscle cells [32], which was typically expressed in male. In the present study, the *Myh11-CreER^T2^; Rosa26-LSL-Tdtomato* transgenic mice were used to label VSMCs. The Myh11-positive cells expressed tdtomato protein [a kind of red fluorescence protein (RFP)]. The total meniscectomy of medial meniscus was performed at 11 d from the start of tamoxifen induction. The knee joints were collected after 4 weeks (Fig. 1A). The validation of using *Myh11-CreER^T2^; Rosa26-LSL-Tdtomato* transgenic mice to trace smooth muscle cell lineage was confirmed. In the transgenic mice tail tissue, the RFP signals were restricted within the blood vessels (Fig. 1B). The meniscus-like tissue sprouted between femur and tibia cartilage. The robust expression of chondrogenic markers (SOX9 and COL II) was presented in the RFP-positive cells, further confirming the transdifferentiation of VSMCs into chondrocyte-like cells (Fig. 1C). In native healthy meniscus of lineage tracing transgenic mice, some RFP-positive cells also colocalized with SOX9 signals (Fig. S1). SM22α is another identified marker of VSMCs [33], which was expressed in male and female. Additionally, the focal full-thickness meniscal defect model was prepared in the avascular zone of medial meniscus anterior horn of female rabbit (Fig. 1D). The robust costaining of α-smooth muscle actin (α-SMA), SM22α, and SOX9 was observed in the repaired tissue section of rabbit meniscus. The robust costaining of α-SMA, SM22α, and COL II was also observed in the regenerated tissue (Fig. 1E), demonstrating that VSMCs transdifferentiated into chondrocyte-like cells and contributed to meniscal healing in the rabbit meniscus defect model. The tissue slices incubated with only secondary antibodies (donkey anti-goat IgG H&L–Alexa Fluor 488, donkey anti-mouse IgG H&L–Alexa Fluor 594, donkey anti-rabbit IgG H&L–Alexa Fluor 647) were used as negative controls (Fig. S2).

### VSMC transplantation facilitated meniscal repair in a rabbit meniscus focal defect model

In order to investigate the efficacy of VSMC transplantation on meniscal repair, the VSMCs were encapsulated with gel-MA hydrogel and then transplanted to meniscal focal defect of the rabbit model (Fig. S3A). Moreover, in order to trace the fate of transplanted cells, the VSMCs from male rabbits were transplanted to female rabbits. Then, the ISH of male-specific sex-determining region Y-linked (SRY) gene could be used to distinguish transplanted VSMCs. At 12 weeks after implantation, meniscus tissue was harvested and meniscal repair was assessed. In the VSMC transplant group, the repaired tissue contained more cell clusters with FC-like cell morphology. The extracellular matrix (ECM) in repaired tissue demonstrated robust deposition of glycosaminoglycans (GAGs) reflected by safranin O staining, COL I, and COL II. Moreover, the repaired tissue demonstrated superior integration with peripheral native meniscus tissue. However, in the blank gel-MA hydrogel group, inferior integration and matrix deposition was demonstrated. The VSMC transplant group demonstrated higher meniscal repair scores (Fig. S3B and C). Subsequently, the immunofluorescent costaining of ISH for the SRY gene and chondrogenic marker (SOX9) confirmed the transdifferentiation of transplanted VSMCs into chondrocyte-like cells (Fig. S3D).

### LIPUS enhanced transdifferentiation of rat and rabbit VSMCs into chondrocyte-like cells

**Fig. 2.**
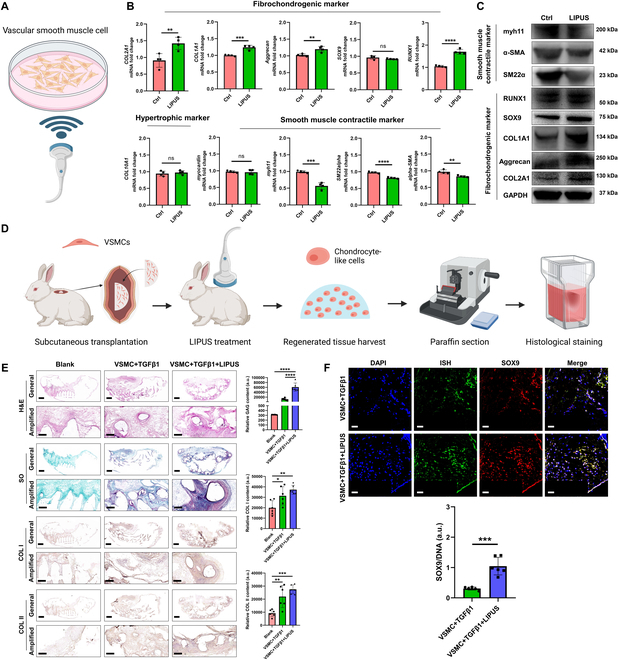
LIPUS initiates the transdifferentiation of VSMCs into chondrocyte-like cells. (A) Schematic diagram of LIPUS stimulus on rat VSMCs in vitro. (B) mRNA levels of the fibrochondrogenic marker, the hypertrophic marker, and the smooth muscle contractile marker in rat VSMCs after LIPUS treatment. *n* = 4, unpaired *t* test. (C) Protein levels of the fibrochondrogenic marker and the smooth muscle contractile marker in rat VSMCs after LIPUS treatment. (D) Schematic diagram demonstrated the transplantation of male rabbit VSMCs into porous scaffold and implanted subcutaneously in the back of female rabbit, followed by LIPUS stimulus. Nine female rabbits (6 months) were used. The blank scaffold, scaffold + VSMCs + TGFβ1, and scaffold + VSMCs + TGFβ1 + LIPUS groups were included. Each group contained 3 rabbits (6 samples). All samples were harvested for histological analysis at 12 weeks after surgery. (E) Histological and immunohistochemical analyses of ectopic fibrocartilage tissue formation after subcutaneous implantation of male rabbit VSMCs. Scale bar of general, 2 mm; scale bar of amplified, 0.5 mm. *n* = 6, one-way ANOVA, *P < 0.05, **P < 0.01, ***P < 0.005, ****P < 0.001. (F) Evaluation of transdifferentiation of transplanted male rabbit VSMCs into chondrocyte-like cells after TGFβ1 or supplemented with LIPUS treatment, which was demonstrated by the costaining of ISH for SRY gene and SOX9 immunofluorescence. Scale bar, 50 μm. *n* = 6, unpaired *t* test, ***P < 0.005.

This part mainly demonstrated that LIPUS could enhance transdifferentiation of VSMCs into chondrocyte-like cells. First, the flow cytometry analysis of isolated rat VSMCs based on the SM22α marker demonstrated a purity of 99.39% (Fig. S4). The rat VSMCs were treated with LIPUS in vitro (Fig. 2A). The fibrochondrogenic, hypertrophic, and smooth muscle contractile markers were evaluated by qPCR and Western blot. After LIPUS treatment, the mRNA levels of fibrochondrogenic markers, including COL2A1, COL1A1, aggrecan, and RUNX1, were up-regulated significantly. However, the chondrogenic marker of SOX9 was not observed to up-regulate in the mRNA level. During chondrogenic transdifferentiation of VSMCs, the mRNA level of hypertrophy, COL10A1 was not up-regulated. However, the smooth muscle contractile markers, including Myh11, SM22α, and α-SMA, were down-regulated significantly in the mRNA level after LIPUS treatment. No significant change of the mRNA level was observed in another smooth muscle contractile marker, myocardin (Fig. 2B). As demonstrated by Western blot, the protein levels of fibrochondrogenic markers (COL2A1, aggrecan, COL1A1, SOX9, and RUNX1) were up-regulated significantly after LIPUS treatment, while the protein levels of smooth muscle contractile markers (SM22α, α-SMA, and Myh11) were down-regulated significantly (Fig. 2C). Second, we identified that LIPUS treatment facilitated chondrogenic transdifferentiation of rabbit VSMCs and consequent ectopic fibrocartilage tissue formation in vivo. For the VSMC + TGFβ1 + LIPUS group, the 3D-printed porous meniscal scaffold was put in the matched mold. The rabbit VSMCs were suspended in gel-MA hydrogel containing 10 ng/ml TGFβ1 and then injected into the porous scaffold, followed by crosslinking with blue light. The porous scaffold was implanted subcutaneously in the back of rabbit followed by LIPUS treatment for 12 weeks (Fig. 2D). For the VSMC + TGFβ1 group, the LIPUS treatment was absent. For the blank group, the blank porous scaffold was implanted subcutaneously. At 12 weeks, the samples were harvested and analyzed histologically. The VSMC + TGFβ1 + LIPUS group demonstrated the highest content of fibrocartilage matrix, including GAG, COL I, and COL II, compared to the VSMC + TGFβ1 group and the blank group (Fig. 2E). In order to distinguish the implanted cells, the VSMCs from male rabbits were transplanted to female rabbits. Identically, using ISH for SRY gene could identify transplanted VSMCs. We performed the immunofluorescent costaining of ISH for SRY gene and SOX9 in the formed tissue, further confirming the chondrogenic transdifferentiation of VSMCs. Moreover, more robust SOX9 expression was observed in the VSMC + TGFβ1 + LIPUS group (Fig. 2F). Third, we identified that LIPUS could enhance VSMC migration in vivo. The 3D-printed porous scaffold was implanted subcutaneously in the back of rat. For the experimental group, LIPUS treatment was performed for 12 weeks (Fig. S5A). Then, the scaffold was harvested and analyzed histologically. Hematoxylin and eosin (H&E) staining demonstrated more cell adhesion in the scaffold. Moreover, more robust expression of SM22α, the marker of VSMCs, was observed within the scaffold of the LIPUS treatment group. Interestingly, in the blank group, SM22α was restricted in the blood vessels, while the SM22α signal was dispersed in the intravascular and extravascular regions in the LIPUS treatment group. This phenomenon demonstrated that LIPUS could enhance emigration of smooth muscle cells out of blood vessels (Fig. S5B1 and B2). Moreover, in the blank group, the SM22α signal was restricted in the blood vessels, which validated the use of SM22α as the VSMC marker (Fig. S5C).

### The mechanisms of LIPUS stimulus on facilitating chondrogenic transdifferentiation of rat VSMCs

**Fig. 3.**
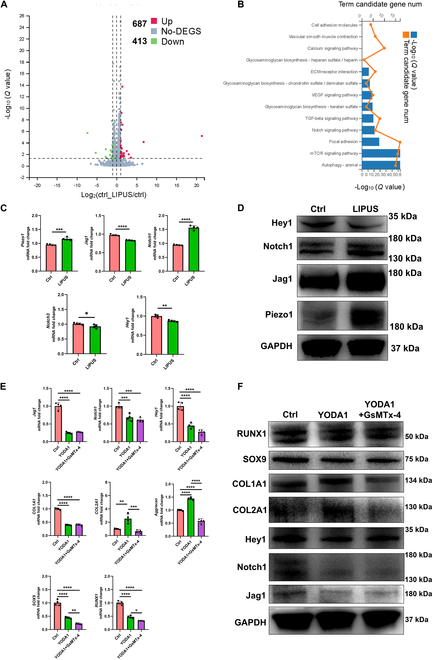
LIPUS facilitates chondrogenic transdifferentiation of rat VSMCs by up-regulating Piezo1 and inhibiting Notch pathway. (A) Volcano plot of differentially expressed genes in rat VSMCs between the control and LIPUS treatment group. (B) KEGG pathway enrichment in rat VSMCs after LIPUS treatment. (C) mRNA levels of Piezo1 and Notch pathway-related genes of rat VSMCs after LIPUS treatment. *n* = 4, unpaired *t* test, *P < 0.05, **P < 0.01, ***P < 0.005, ****P < 0.001. (D) Relative protein levels of Piezo1 and Notch pathway-related markers of rat VSMCs after LIPUS treatment. (E) mRNA levels of rat VSMCs after YODA1 or GsMTx-4 treatment. *n* = 4, one-way ANOVA, *P < 0.05, **P < 0.01, ***P < 0.005, ****P < 0.001. (F) Protein levels of rat VSMCs after YODA1 or GsMTx-4 treatment.

**Fig. 4.**
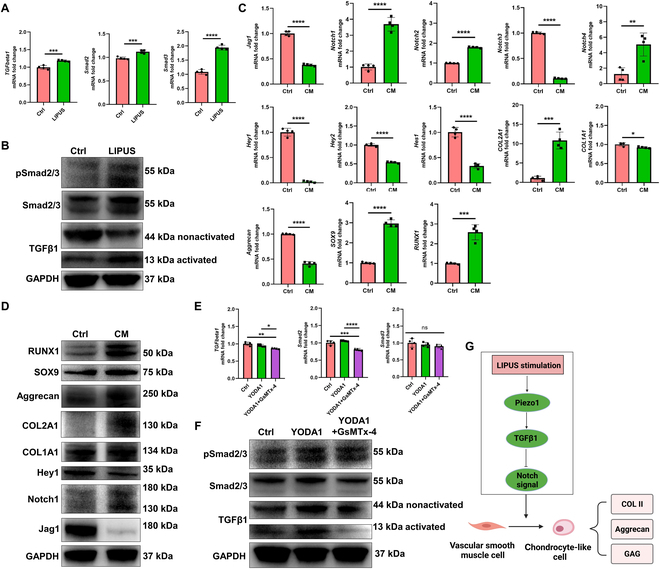
LIPUS facilitates chondrogenic transdifferentiation of rat VSMCs through Piezo1/TGFβ1/Notch axis. (A) mRNA levels of TGFβ1 signaling of rat VSMCs after LIPUS treatment. *n* = 4, unpaired *t* test, ***P < 0.005, ****P < 0.001. (B) Protein levels of TGFβ1 signaling of rat VSMCs after LIPUS treatment. (C) mRNA levels of fibrochondrogenic and Notch pathway-related markers of rat VSMCs after TGFβ1 treatment. *n* = 4, unpaired *t* test, *P < 0.05, **P < 0.01, ***P < 0.005, ****P < 0.001. (D) Protein levels of fibrochondrogenic and Notch pathway-related markers of rat VSMCs after TGFβ1 treatment. (E) mRNA levels of TGFβ1 signaling of rat VSMCs after YODA1 or GsMTx-4 treatment. *n* = 4, one-way ANOVA, *P < 0.05, **P < 0.01, ***P < 0.005, ****P < 0.001, ns represents no significance. (F) Protein levels of TGFβ1 signaling of rat VSMCs after YODA1 or GsMTx-4 treatment. (G) Schematic diagram of molecular mechanisms of LIPUS stimulus on facilitating chondrogenic transdifferentiation of VSMCs.

This part mainly demonstrated that LIPUS stimulus could up-regulate mechanosensitive ion channel Piezo1 expression, then activating the TGFβ1 signal. Upon TGFβ1 signal activation, the Notch signal was inhibited, consequently leading to chondrogenic transdifferentiation of rat VSMCs. First, to identify the underlying changes of gene expression pattern and signal pathway, we performed RNA-sequencing analyses in rat VSMCs after LIPUS treatment in vitro. The genes were differentially expressed after LIPUS treatment (Fig. 3A). Moreover, the Kyoto Encyclopedia of Genes and Genomes (KEGG) pathway analyses revealed that calcium signaling pathway, TGFβ signaling pathway, and Notch signaling pathway were involved (Fig. 3B). Second, we identified that LIPUS stimulus could up-regulate Piezo1 expression, then inhibiting the Notch signal, consequently initiating chondrogenic transdifferentiation of VSMCs. LIPUS could provide acoustic mechanical stimulation, which inspired us to focus on mechanics-sensing molecules as downstream effectors. The mechanosensitive ion channel Piezo1 was the best characterized biological force-sensing system [34]. Previous studies demonstrated that Piezo1 mediated the transformation of ultrasound acoustic mechanical stimulus into biochemical effect [13]. The mRNA and protein levels of Piezo1 were up-regulated significantly in rat VSMCs after treatment with LIPUS (Fig. 3C and D). The KEGG pathway enrichment analysis demonstrated that the Notch signaling pathway was involved during chondrogenic transdifferentiation of VSMCs. The activation of Notch signaling played a critical role in maintaining contractile phenotype of VSMCs, while the chondrogenic transdifferentiation would be initiated upon repression of the Notch signal [11]. After LIPUS treatment, the mRNA level of Notch ligand (Jag1) was down-regulated significantly. The mRNA level of Notch receptor (Notch1) was up-regulated significantly, while the mRNA level of Notch receptor (Notch3) was down-regulated significantly. The downstream target effector of the Notch signal (Hey1) was down-regulated significantly, demonstrating repression of the Notch signal. The protein levels of Notch1 and Hey1 were consistent with the mRNA level, demonstrating Notch1 up-regulation and Hey1 down-regulation (Fig. 3C and D). However, the protein level of Jag1 was up-regulated significantly after LIPUS treatment, which may be attributed to different chondrogenic transdifferentiation stages [35]. The up-regulation of the Jag1 and Notch1 protein level was contradictory to the down-regulation of Hey1, which may be attributed to the complexity of the Jag/Notch signal. The Jag/Notch signal contained 5 kinds of ligands (Jag1, Jag2, Dll1, Dll3, and Dll4) and 4 kinds of receptors (Notch1, Notch2, Notch3, and Notch4) [36]. However, the downstream target effector Hey1 could reflect the activation degree of the Notch signal. Until now, we have identified that LIPUS could up-regulate Piezo1 expression and repressed the Notch signal. In order to further elucidate the relation between the Piezo1 and Notch signal, the Piezo1 agonist (YODA1) and antagonist (GsMTx-4) were applied. After YODA1 treatment, the typical fibrochondrogenic marker (COL1A1 and COL2A1) was up-regulated significantly in the protein level, although the chondrogenic transcriptional factor (SOX9 and RUNX1) was not up-regulated, while the addition of GsMTx-4 repressed the effect of YODA1 on chondrogenic transdifferentiation, demonstrating down-regulation of COL2A1. Especially, the YODA1 treatment repressed the Notch signal, demonstrating down-regulation of Jag1, Notch1, and Hey1, while the addition of GsMTx-4 reversed the effect of YODA1 on the Notch signal in some degree, demonstrated by up-regulation of Hey1 (Fig. 3E and F). Additionally, in order to confirm the direct evidence that LIPUS promoted VSMC transdifferentiation into chondrocyte-like cells through Piezo1, GsMTx-4 was added during LIPUS treatment. The immunofluorescent results demonstrated that LIPUS treatment promoted VSMC transdifferentiation into chondrocyte-like cells, demonstrating down-regulation of α-SMA and SM22α and up-regulation of SOX9 and COL II, while GsMTx-4 blocked LIPUS effects (Fig. S6). Therefore, LIPUS stimulus up-regulated Piezo1 expression, then repressing the Notch signal, consequently initiating chondrogenic transdifferentiation of VSMCs. Third, we identified that LIPUS activated the TGFβ1 signal, then inhibiting the Notch signal, consequently initiating chondrogenic transdifferentiation of VSMCs. As aforementioned, the KEGG enrichment analysis revealed that the TGFβ signaling pathway was involved after LIPUS treatment. Previous studies demonstrated that the elevated activation of TGFβ signaling was a critical modulator driving the chondrogenic transdifferentiation of VSMCs [37]. After LIPUS treatment, the mRNA levels of TGFβ1, smad2, and smad3 were up-regulated significantly (Fig. 4A). Moreover, the protein levels of activated TGFβ1, smad2/3, and phosphorylated smad2/3 (pSmad2/3) were up-regulated (Fig. 4B). However, the protein level of non-activated TGFβ1 decreased significantly, which could be explained by the intrinsic feedback regulation of the TGFβ signal [38]. After treatment with chondrogenic medium containing TGFβ1 (10 ng/ml), the Jag/Notch signal was repressed significantly, demonstrating down-regulation of Jag1, Notch3, and downstream target effectors (Hey1, Hey2, and Hes1). The fibrochondrogenic markers, including COL1A1, COL2A1, aggrecan, SOX9, and RUNX1, were up-regulated significantly (Fig. 4C and D). Therefore, we confirmed that LIPUS activated the TGFβ1 signal, then repressing the Notch signal, consequently enhancing chondrogenic transdifferentiation of VSMCs. Lastly, to identify the relation between the Piezo1 and TGFβ1 signal, the Piezo1 agonist (YODA1) and antagonist (GsMTx-4) were applied. The YODA1 treatment activated the TGFβ1 signal, while GsMTx-4 reversed the effect (Fig. 4E and F). Therefore, Piezo1 was the upstream of the TGFβ1 signal. Thus, we identified that LIPUS stimulus up-regulated Piezo1 expression, then activating the TGFβ1 signal. Upon TGFβ1 signal activation, the Notch signal was repressed, consequently initiating chondrogenic transdifferentiation of VSMCs (Fig. 4G).

### 3D printing porous scaffold combining LIPUS stimulus facilitated meniscal fibrocartilage regeneration in a beagle canine subtotal meniscectomy model

**Fig. 5.**
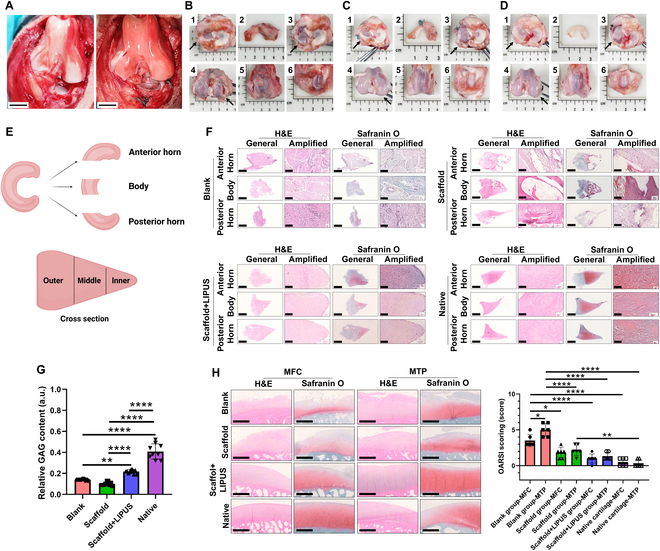
The macroscopic and histological analyses of regenerated tissue in beagle canine at 6 months. (A) Preparation of subtotal meniscectomy (left) and PU_PCL porous scaffold implantation (right) in beagle canine. Scale bar, 1 cm. (B) Macroscopic appearance of the blank group (1, the macroscopic appearance in tibia, black arrowhead represents medial side; 2, the regenerated tissue after subtotal meniscectomy; 3, cartilage status in MTP, black arrowhead represents medial side; 4, cartilage status in MFC, black arrowhead represents medial side; 5, cartilage status in trochlea; 6, cartilage status in patella). (C) Macroscopic appearance of the scaffold group. (D) Macroscopic appearance of the scaffold + LIPUS group. (E) Schematic diagram of division of meniscus. (F) Histological analyses of regenerated tissue and native meniscus in beagle canine at 6 months. Scale bar of general, 2 mm; scale bar of amplified, 100 μm. (G) Semiquantitative analysis of GAG within regenerated tissue and native meniscus. *n* = 9, one-way ANOVA, **P < 0.01, ****P < 0.001. (H) Histological analysis and OARSI scoring of cartilage degeneration. Scale bar, 1 mm. *n* = 6, one-way ANOVA, *P < 0.05, **P < 0.01, ****P < 0.001.

The subtotal meniscectomy model of medial meniscus was prepared in the beagle canine. Then, the 3D printing porous PU_PCL scaffold was implanted (Fig. 5A). From macroscopic view, for the blank group, only some fibrous tissues adhered to the peripheral residual meniscal tissue. Apparent synovitis was observed in the whole knee joint. Obvious cartilage erosion, defect, and osteophytes could be observed, especially in MFC and MTP (Fig. 5B). For the scaffold group, the morphology of newly formed tissue resembled that of native meniscus. Most part of the scaffold was covered with mixed fibrous and fibrocartilage-like white tissue. The degree of joint synovitis was milder than that of the blank group. However, moderate cartilage erosion and some osteophytes were observed in MTP (Fig. 5C). For the scaffold + LIPUS group, the regenerated tissue was more like native meniscus. The scaffold was enwrapped with fibrocartilage-like tissue. No visible synovitis could be observed. The mild abrasion was observed in MTP cartilage (Fig. 5D). The sample was divided into anterior horn, body, and posterior horn for subsequent histological analyses. Moreover, the tissue section was classified as inner, middle, and outer zone from the inner edge to the peripheral outer edge (Fig. 5E). In the scaffold + LIPUS group, the histological assessment demonstrated the formation of fibrocartilaginous tissue, showing abundant collagen and proteoglycan matrix deposition. The regenerated tissue was rich in oval to round chondrocyte-like cells. The safranin O staining demonstrated that the regenerated tissue was rich in GAG, mainly in the inner and middle zone like native meniscus. In contrast, for the blank group, the histological analysis demonstrated fibrous tissue. Almost no GAG content was deposited, demonstrating very light safranin O staining. In the scaffold group, the regenerated tissue demonstrated mixed fibrous and fibrocartilaginous tissue. The safranin O staining demonstrated some degree of GAG deposition. The regenerated tissue demonstrated poor integration with residual native meniscus tissue (Fig. 5F). The GAG content analysis demonstrated that the scaffold + LIPUS group possessed the richest GAG deposition compared to that of the blank and scaffold group, and was closer to native meniscus (Fig. 5G). The cartilage histology demonstrated that the scaffold + LIPUS group showed less cartilage abrasion, GAG depletion, and lower OARSI cartilage degeneration scores compared to the blank and scaffold group. However, in the blank group, severe cartilage erosion or defect and GAG loss could be observed, especially in MTP. The blank group possessed the highest OARSI scores. In the scaffold group, moderate cartilage abrasion and GAG depletion was observed (Fig. 5H).

The repaired tissue was rich in COL I and COL II content in the scaffold + LIPUS group. The COL II content within regenerated tissue was mainly in the inner and middle zone. The whole regenerated tissue was abundant in COL I, but was mainly in the outer zone. However, less COL I and COL II deposition was observed in the blank and scaffold group (Figs. S7 to S10). The semiquantitative analysis demonstrated that the scaffold + LIPUS group possessed the highest content of COL I and COL II compared with that of the blank and scaffold group, and was closer to native meniscus (Fig. S11). Next, the aggrecan matrix deposition was evaluated. The regenerated tissue in the scaffold + LIPUS group contained abundant aggrecan matrix, which was mainly in the inner and middle zone like native meniscus. However, very little aggrecan matrix was observed in the blank and scaffold group, compared to that of the scaffold + LIPUS group and native menisci (Figs. S12 to S15). The semiquantitative analysis demonstrated that the scaffold + LIPUS group possessed the highest content of aggrecan compared to that of the blank and scaffold group, and was closer to native menisci (Fig. S16). Previous studies have demonstrated that pericellular matrix (perlecan and type 6 collagen, COL VI) was critical to meniscal FCs, such as the mechanobiology [39]. Then, the pericellular matrix of regenerated tissue was characterized. The immunofluorescent costaining of perlecan and COL VI demonstrated that the regenerative cells in the scaffold + LIPUS group contained abundant pericellular matrix, while very less pericellular matrix was observed in the blank and scaffold group (Fig. S17A and B). The semiquantitative analysis demonstrated that the scaffold + LIPUS group possessed the highest content of perlecan and COL VI compared to the blank and scaffold group (Fig. S17C and D). A previous study demonstrated that lysyl oxidase (LOX) was the main enzyme, which catalyzed collagen crosslink [40]. The collagen fiber crosslink density affected the mechanical strength of menisci [41]. The scaffold + LIPUS group demonstrated the highest content of LOX compared to that of the blank and scaffold group (Fig. S18). The second harmonic generation (SHG) signals generated by collagen fibers under 2-photon microscopy were associated with collagen matrix. The content of collagen matrix in the scaffold + LIPUS group was the highest compared with that of the blank and scaffold group, and resembled that of native meniscus (Fig. S19). In addition, the elastic modulus and hardness were evaluated by nanoindentation test (Fig. S20A). The elastic modulus of the scaffold + LIPUS group (17.96 ± 8.40 MPa) was superior to that of the blank group (0.18 ± 0.04 MPa) and the scaffold group (0.73 ± 0.14 MPa), but was still inferior to that of native meniscus (56.26 ± 17.78 MPa) significantly (Fig. S20B). The hardness of the scaffold + LIPUS group (1.52 ± 0.20 MPa) was superior to that of the blank group (0.06 ± 0.02 MPa) and the scaffold group (0.60 ± 0.22 MPa), but was still inferior to that of native meniscus (2.80 ± 0.82 MPa) significantly (Fig. S20C). We then evaluated the expression of Piezo1, TGFβ1, Jag1, Notch1, and Hey1 in the regenerated tissue of beagle canine. LIPUS stimulus enhanced the expression of Piezo1 and TGFβ1 significantly during meniscal regeneration in vivo (Figs. S21 and S22). For the Notch pathway-related markers, after LIPUS treatment, Jag1 expression was significantly decreased (Fig. S23) and Notch1 expression was significantly increased (Fig. S24). However, the downstream target effector of the Notch pathway (Hey1) was decreased significantly (Fig. S25), which represented Notch pathway depression. Moreover, the fibrochondrogenic transdifferentiation of VSMCs was identified in the regenerated meniscus tissue of the scaffold + LIPUS group, demonstrating colocalization of the VSMC marker (SM22α and Myh11) and the fibrochondrogenic marker (SOX9, COL I, and COL II) (Figs. S26 and S27).

### The scRNA-Seq analyses of regenerated meniscal tissue in the beagle canine

**Fig. 6.**
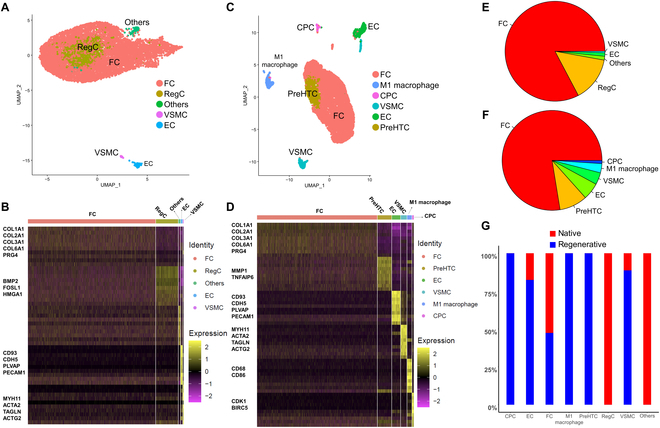
The single-cell atlas of beagle canine native and regenerated meniscal tissue. (A) UMAP demonstrates a total of 5 cell clusters identified in native beagle canine meniscus, which contain 11,523 cells. (B) Heatmap of differentially expressed genes in the 5 cell clusters. (C) UMAP demonstrates a total of 6 cell clusters identified in regenerated beagle canine meniscus after LIPUS treatment, which contain 10,891 cells. (D) Heatmap of differentially expressed genes in the 6 cell clusters. (E) Proportion of each cell cluster in native meniscus. (F) Proportion of each cell cluster in regenerated meniscus. (G) Combined proportion of each cell cluster in native and regenerated meniscal tissue.

To identify the cellular composition and differences between the regenerated meniscal tissue in the scaffold + LIPUS group and the native beagle canine meniscal tissue, the transcriptomes of individual live cells were isolated and sequenced using scRNA-Seq. A total of 11,523 and 10,891 cells were isolated in the native and regenerated meniscal tissue, respectively (Table S5). The cell quality met the requirement for subsequent single-cell sequencing (Fig. S28). We identified 5 major cell populations in the native meniscal tissue and 6 major cell populations in the regenerated meniscal tissue. Concretely, the 5 major cell populations in the native meniscal tissue were as follows: (a) FCs (expressing COL1A1, COL2A1, COL3A1, COL6A1, and PRG4) [42,43], (b) regulatory chondrocytes (RegCs; expressing BMP2, FOSL1, and HMGA1) [44], (c) VSMCs (expressing Myh11, ACTA2, TAGLN, and ACTG2) [45,46], (d) endothelial cells (ECs; expressing CD93, CDH5, PLVAP, and PECAM1) [47,48], and (e) one unidentified cell cluster (Others) (Fig. 6A and B). The 6 major cell populations in the regenerated meniscal tissue after LIPUS treatment were as follows: (a) FCs (expressing COL1A1, COL2A1, COL3A1, COL6A1, and PRG4), (b) prehypertrophic chondrocytes (PreHTCs; expressing MMP1 and TNFAIP6) [49], (c) cartilage progenitor cells (CPCs; expressing CDK1 and BIRC5) [42], (d) VSMCs (expressing Myh11, ACTA2, TAGLN, and ACTG2), (e) ECs (expressing CD93, CDH5, PLVAP, and PECAM1), and (f) M1 macrophage (expressing CD68 and CD86) [50] (Fig. 6C and D). No matter in the native meniscal tissue or the regenerated meniscal tissue, the FCs were the main cell type. The proportion of VSMC and EC in the regenerated meniscal tissue increased compared to that of the native meniscal tissue. Moreover, the CPC, PreHTC, and M1 macrophage emerged in the regenerated meniscal tissue, but were absent in native meniscus (Fig. 6E to G).

**Fig. 7.**
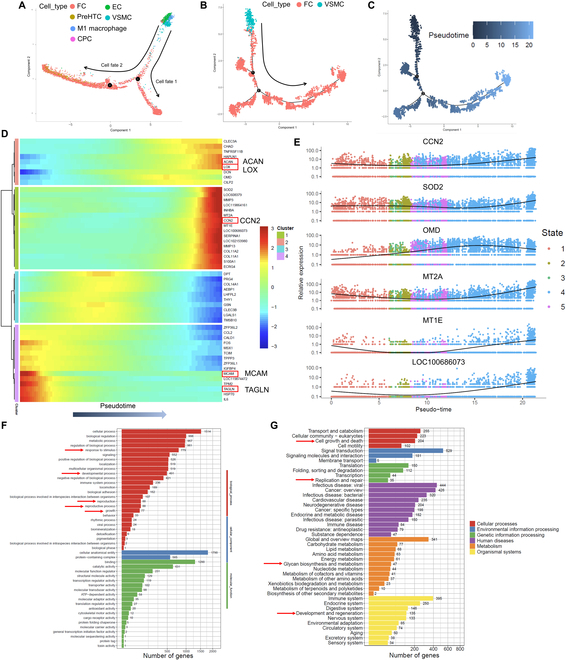
The pseudotime trajectory analysis of cell clusters and GO and KEGG pathway enrichment analysis in beagle canine regenerated meniscal tissue. (A) Monocle pseudotime trajectory showing the progression of VSMC, EC, M1 macrophage, FC, CPC, and PreHTC. (B) Monocle pseudotime trajectory of VSMC and FC. (C) Pseudotime between VSMC and FC. (D) Heatmap of top 50 significant genes in pseudotime trajectory of VSMC and FC. (E) Top 6 significant differentially expressed genes in pseudotime trajectory of VSMC and FC. (F) GO analysis of all cells within regenerated tissue. (G) KEGG pathway enrichment analysis of all cells within regenerated tissue.

Subsequently, we investigated the differentiation trajectory among cell clusters in the regenerated meniscal tissue of the scaffold + LIPUS group. The trajectory contained 2 termini, which corresponded to 2 cell fates. The root of the trajectory was mainly composed of VSMC and EC. The 2 termini were populated with FC in cell fate 1 and FC and PreHTC in cell fate 2 (Fig. 7A). Combining the results of trajectory analyses and the aforementioned in vitro and in vivo results, VSMCs were identified to transdifferentiate into FCs within the regenerated meniscal tissue. Next, the VSMC and FC were selected to form a new trajectory to study gene expression changes during transdifferentiation (Fig. 7B and C). The expression of the smooth muscle contractile marker (TAGLN, also known as SM22α) decreased. The expression of progenitor index (MCAM, also known as CD146) [51] in VSMC down-regulated during transdifferentiation. The expression of CCN2 (also known as CTGF) was up-regulated in VSMC during trandifferentiation. CCN2 played an important role in the synthesis of ECM, especially in musculoskeletal tissue such as meniscus, cartilage, and intervertebral disc [52]. Moreover, the expression of ACAN (aggrecan, a proteoglycan rich in native meniscus tissue) was up-regulated. The expression of the collagen crosslinking enzyme LOX was up-regulated during fibrochondrogenic transdifferentiation (Fig. 7D). Next, the top 6 significantly differentially expressed genes during transdifferentiation were identified, including CCN2, SOD2 (superoxide dismutase 2), OMD (osteomodulin), MT2A (metallothionein 2A), MT1E (metallothionein 1E), and LOC100686073. All the 6 genes were up-regulated (Fig. 7E). The GO analysis demonstrated that response to stimulus, developmental process, reproduction, reproductive process, and growth were involved in the regenerative process of the meniscal tissue (Fig. 7F). The KEGG pathway enrichment analysis demonstrated that cell growth and death, replication and repair, glycan biosynthesis and metabolism, and development and regeneration were involved in the regenerative process of meniscus (Fig. 7G). Moreover, within the context of the scRNA-Seq data of the regenerated meniscal tissue of beagle canine after LIPUS treatment, the expression patterns of the Notch signaling pathway (Notch1, Notch3, Jag1, and Hey1) were down-regulated from VSMC to FC (Fig. S29).

## Discussion

3D printing technology has been widely applied in meniscus tissue engineering due to its precise manufacturing of scaffold morphology and inner structures. The repair strategies for meniscus repair and regeneration using 3D printing innovated gradually in several aspects. The first aspect was scaffold manufacturing methods. In the early phase, the porous meniscus scaffold was mainly prepared using layer-by-layer extrusion-based 3D printing [7,8,22,53]. As the development of 3D printing technology, the DLP (digital light processing) printing technology was applied gradually to manufacture meniscus scaffold [54]. The second aspect was the development of new materials for 3D printing meniscus scaffold. PCL was the most predominant material for meniscus scaffold manufacture in the early phase [7,8,22]. However, the rigid property of PCL material hindered its clinical translation for meniscus repair in the future. Thus, new materials have been developed, which were friendly to articular cartilage. For example, one previous study developed a PU-based elastomer–hydrogel composite meniscal scaffold with biomimetic gradient structure and robust interface to repair meniscus and prevent cartilage degeneration [55]. The third aspect was the addition to the 3D-printed meniscus scaffold. Previous studies have added growth factors to the meniscus scaffold, such as TGFβ and CTGF [8], which were expected to drive chondrogenesis of stem cells. Later, the small molecule, such as KGN, was added into the scaffold to facilitate chondrogenic matrix production [54,55]. The bioink from decellularized natural meniscus matrix was also applied to the meniscus scaffold to facilitate meniscal repair [56]. Finally, the repair cells varied greatly. Previous studies have encapsulated exogenous MSCs into the 3D-printed scaffold to repair meniscus [7]. However, how to facilitate the repair capacity of endogenous repairable cells during meniscus regeneration lacked studies. Herein, we demonstrated the critical role of migrated VSMCs in meniscal fibrocartilage regeneration and the effect of LIPUS stimulus on chondrogenic transdifferentiation of VSMCs.

First, we identified VSMCs transdifferentiated into chondrocyte-like cells and participated in meniscal regeneration. VSMCs are highly specialized and differentiated in adults. The primary role of VSMCs is to contract, which controls vessel tone and diameter. Normally, VSMCs proliferate at a low rate and demonstrate low activity of synthesis. However, mature VSMCs are not terminally differentiated and maintains remarkable phenotypic plasticity. The phenotypic switching of VSMCs is likely initiated by variations of environmental cues or extracellular signals. Moreover, during embryogenesis and blood vessel remodeling, the phenotypic plasticity of VSMCs is also required for blood vessel formation and maturation [57,58]. Recently, VSMC phenotypic switching has been clarified to participate in the pathogenesis of atherosclerosis. A previous study using the VSMC lineage tracing model demonstrated the evidence that VSMCs transdifferentiated into osteochondrogenic precursors and chondrocyte-like cells in the calcified vessels of matrix Gla protein-deficient (MGP^−/−^) mice [59]. The enhanced activation of the TGFβ signal has been shown to be a critical modulator initiating the chondrogenic transdifferentiation of VSMCs [37,60]. TGFβ cytokine has been shown to be secreted by synovium and cartilage and replenished into the synovial fluid continuously [61]. Moreover, the mature TGFβ peptide could be activated by mechanical shearing stimulus exerted by the joint motion [62]. Thus, it was reasonable to hypothesize that VSMCs transdifferentiated into chondrocyte-like cells and contributed to meniscal cartilage regeneration. In the present study, the meniscectomy model of VSMC lineage tracing transgenic mice confirmed the transdifferentiation of VSMCs into chondrocyte-like cells during the intrinsic meniscal regeneration process. Furthermore, within the regenerated tissue of meniscus defect in rabbit, the VSMCs demonstrated robust expression of SOX9 and COL II, the typical chondrogenic marker. Thereby, enhancing the transdifferentiation of VSMCs into chondrocyte-like cells to secrete cartilaginous matrix could be a novel way to facilitate meniscal cartilage regeneration.

Second, we demonstrated that LIPUS stimulus enhanced the transdifferentiation of VSMCs into chondrocyte-like cells and facilitated meniscal fibrocartilage regeneration in vivo. LIPUS is a form of low-intensity acoustic radiation identified by the pulsed-wave form. The pulse period of LIPUS was composed of ON and OFF signal periods, which were termed as the duty cycle (Fig. S30). The intensity of LIPUS was determined by the amplitude of the ON signal period, which ranged from 0.02 to 1 W/cm^2^ at frequencies of 1 to 3 MHz [14]. No physiologic stress could be induced by LIPUS treatment, with only 0.5 °C increase in temperature after LIPUS treatment for 10 min [63]. LIPUS has been approved by the U.S. FDA for the treatment of fresh bone fractures and non-unions [14]. Recently, the chondrogenic effect of LIPUS has been demonstrated by many previous studies. Nishida et al. [15] demonstrated that LIPUS treatment with 60 mW/cm^2^ at 3.0-MHz frequency for single 20-min session activated phosphorylation of p38 mitogen-activated protein kinase (MAPK) and extracellular signal-regulated kinase 1/2 (ERK1/2) in human chondrocytic cell line-2/8 (HCS-2/8), following up-regulated expression of COL2A1 and aggrecan. Uddin et al. [17] concluded that LIPUS increased the synthesis of proteoglycans in human cartilage explants and suppressed interleukin-1β (IL-1β)-induced proteoglycans loss. In addition to the anabolic effect of LIPUS on chondrocytes and cartilage explants, LIPUS significantly facilitated MSC chondrogenesis. Xia et al. [64] demonstrated that LIPUS enhanced the chondrogenesis of bone marrow-derived MSCs of rat through the integrin–mammalian target of rapamycin (mTOR) signaling pathway. Wang et al. [16] concluded that LIPUS promoted rat MSC chondrogenesis by inhibiting autophagy, demonstrating up-regulated expression of COL2A1, aggrecan, and SOX9. Regarding the remarkable effect of LIPUS on chondrogenesis, it was hypothesized that LIPUS could promote the chondrogenic phenotypes of VSMCs. In the present study, we confirmed that LIPUS stimulus up-regulated chondrogenic markers of rat VSMCs but down-regulated smooth muscle contractile markers in vitro. We also demonstrated LIPUS stimulus facilitated the chondrogenic transdifferentiation of rabbit VSMCs when implanted subcutaneously and enhanced the formation of ectopic meniscus-like fibrocartilage tissue. Especially, the present study confirmed that LIPUS facilitated the migration of VSMCs onto blank scaffold in vivo when implanted subcutaneously in rat, which was consistent with previous studies demonstrating the promoting effect of LIPUS on angiogenesis [65,66]. More importantly, using the subtotal meniscectomy model of beagle canine, more fibrocartilage tissue wrapped the implanted scaffold after regular LIPUS stimulus. The regenerated tissue was mainly populated by oval to round-shaped chondrocyte-like cells. More chondrogenic matrix, including GAG, COL II, and aggrecan, could be observed in the regenerated tissue, especially in the inner and middle zone, demonstrating anisotropic distribution like native menisci. In addition to chondrogenic matrix, the regenerated tissue after LIPUS stimulus demonstrated superior pericellular matrix deposition (perlecan and collagen VI), which was critical for mechanobiology of FCs [67], and collagen crosslink enzyme (LOX) production, which was critical for collagen crosslinking and collagen strength [68]. Moreover, superior mechanical properties enabled the regenerated tissue to withstand more mechanical shock exerted by joint, thus maintaining mechanical homeostasis and protecting cartilage from degeneration. Specially, the immunofluorescent costaining of VSMC markers and fibrochondrogenic markers confirmed the transdifferentiation of VSMCs into FCs within the regenerated meniscal tissue. In the present study, the strategy of using 3D-printed porous scaffold implantation combining LIPUS stimulus, which facilitated fibrochondrogenic transdifferentiation of VSMCs, was effective in promoting meniscal fibrocartilage regeneration and was more translational compared to the traditional meniscal tissue engineering strategy using exogenous cells and biochemical factors [7].

Moreover, the molecular mechanisms of LIPUS stimulus on facilitating chondrogenic transdifferentiation of VSMCs were elucidated. Although previous studies demonstrated that LIPUS stimulus promoted anabolism of chondrocytes by activating the MAPK and ERK1/2 signal [15], or enhanced MSC chondrogenesis by activating the integrin–mTOR signal [64], no previous study elucidated the mechanisms of LIPUS on facilitating chondrogenic transdifferentiation of VSMCs to our knowledge. LIPUS provided acoustic mechanical stimulus, inspiring us to concentrate on mechanics-sensing molecules as downstream effectors. We confirmed that the mechanosensitive Piezo1 molecule in rat VSMCs was up-regulated significantly after LIPUS treatment. This was consistent with previous studies demonstrating that Piezo1 transformed acoustic mechanical stimulus into the biochemical signal [13,69]. A previous study demonstrated that continuous Notch signaling activation was essential to maintain the contractile phenotype of VSMCs and prevent reprogramming of VSMCs. However, in the absence of Notch signaling, VSMCs experienced the chondrogenic transdifferentiation [11]. Beazley et al. [37] concluded that elevated activation of TGFβ signaling repressed the Notch signal and induced chondrogenic transformation of VSMCs. Consistent with the previous study, we confirmed that the activation of TGFβ1 signaling after LIPUS stimulus suppressed the Notch signal and then enhanced chondrogenic transformation of VSMCs. We also identified that Piezo1 was the upstream of the TGFβ1 signal. Herein, we concluded that LIPUS stimulus up-regulated Piezo1 expression, then activated the TGFβ1 signal, following repression of the Notch signal, consequently leading to chondrogenic transdifferentiation of VSMCs.

The scRNA-Seq analyses showed a similar cellular composition between the regenerated meniscal tissue and native meniscus of beagle canine. The FCs were the most prevailing cell population in the regenerated meniscal tissue, which was identical to native meniscus. The proportion of VSMCs and EC in the regenerated meniscal tissue increased compared to that of native meniscus, demonstrating abundant angiogenesis during meniscal regeneration, which was like native fetal meniscus [70]. Combining the results of trajectory analyses and in vitro and in vivo studies, the fibrochondrogenic transdifferentiation of VSMC was confirmed during meniscal regeneration. The smooth muscle contractile-related gene expression decreased, while the FC-related gene expression was up-regulated. The GO and KEGG analyses of scRNA-Seq demonstrated that the developmental and regenerative molecules and pathways were enriched during meniscal regeneration. Interestingly, we found that EC was also located in the root of trajectory. A previous study isolated a population of EC (CD93^+^/MCAM^+^) from native human meniscus. They concluded that this population possessed progenitor properties, demonstrating colony-forming and various cell lineage differentiation capacity [71]. It was speculated that EC may be another origin of FC during meniscal regeneration, like VSMCs. Further studies were needed to study the role of EC in meniscal regeneration. The CPC cell population was observed in the regenerated meniscal tissue, which was characterized by multilineage differentiation capacity, colony-forming ability, and migration ability [72]. Moreover, the CPC was distributed in the 2 branches of trajectory line accompanying FC (Fig. 7A), indicating its role in meniscal regeneration. However, further studies were warranted to investigate the relation between CPC and VSMC, FC. Additionally, we found that M1 macrophage emerged in the regenerated meniscal tissue. This phenomenon may be caused by the degraded PCL scaffold microparticles. The M1 macrophage possessed more robust phagocytic activity of microplastic particles than M2 macrophage [73]. M1 macrophage secreted proinflammatory cytokines [such as IL-6, IL-1β, and tumor necrosis factor-α (TNF-α)] and degradation enzymes [such as matrix metalloproteinase (MMP)], which was adverse for tissue repair, while M2 macrophage secreted anti-inflammatory cytokines, which were beneficial to tissue regeneration [74]. Thus, better meniscal regeneration may be obtained by modulating the M1/M2 macrophage balance in future studies. The scRNA-Seq analyses demonstrated a multiple cellular composition within the regenerated meniscal tissue. The derivation of these cells warranted further investigation, which could provide therapeutic clues for meniscal repair and regeneration. Previous studies have demonstrated that synovial tissue participated in meniscal repair and regeneration [23,75,76]. Synovial tissue contained multiple types of cells, such as fibroblasts, MSCs, and so on [77]. Moreover, abundant blood vessels were present in the synovium [77]. The vascularization within the regenerated meniscal tissue was speculated to be derived from the synovium. If so, enhancing synovial tissue migration to the injured or defect region of meniscus could be beneficial for meniscal repair and regeneration, which necessitated further study.

## Conclusion

**Fig. 8.**
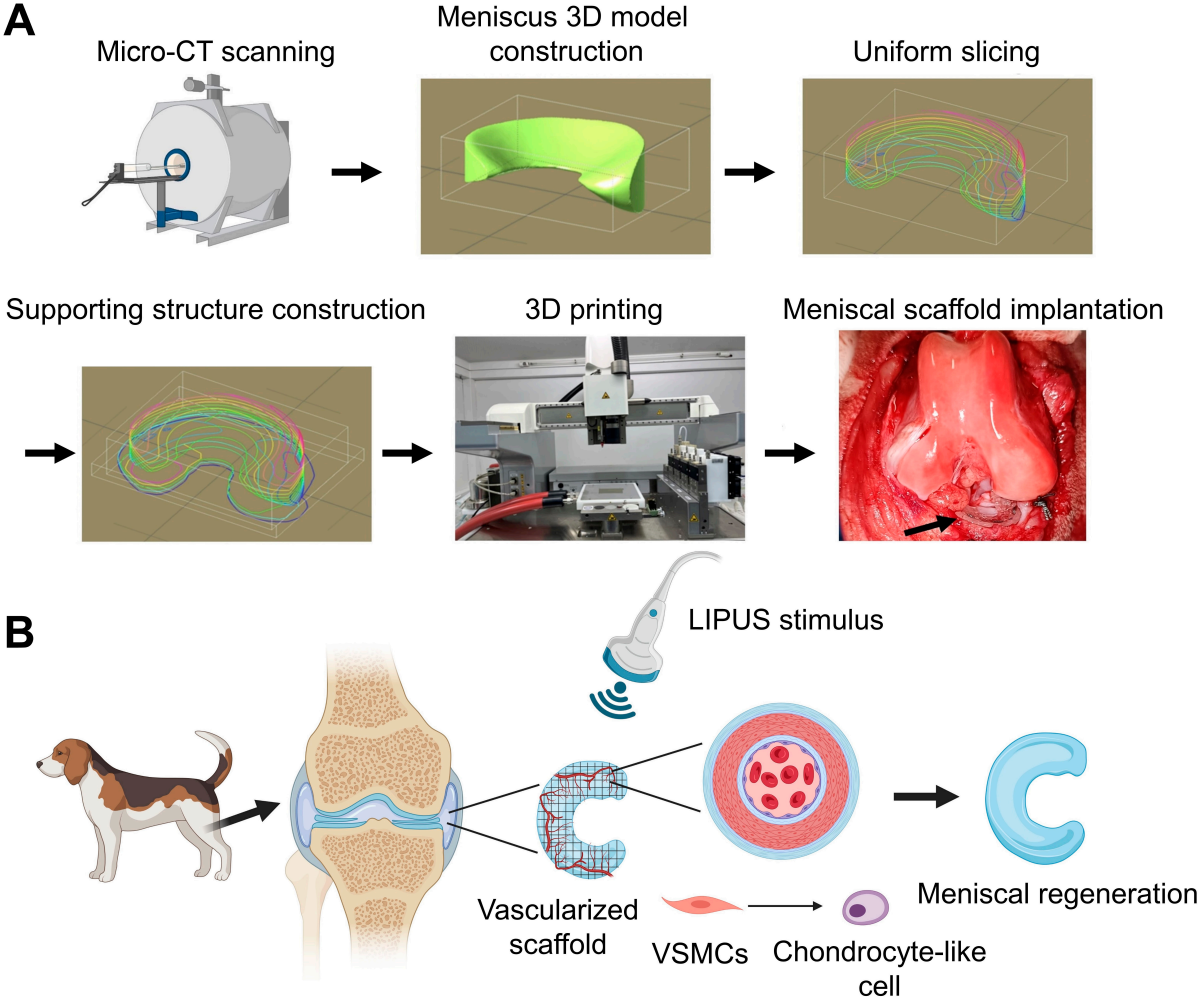
The schematics diagram showing that the combination of PU_PCL scaffold implantation and regular LIPUS stimulus facilitates meniscal fibrocartilage regeneration in a beagle canine subtotal meniscectomy model. (A) Flow diagram of beagle canine meniscal scaffold 3D printing and implantation. (B) Schematic diagram of regular LIPUS stimulus on facilitating VSMC chondrogenic transdifferentiation and consequent meniscal regeneration.

In conclusion, we first identified that VSMCs transdifferentiated into FCs and participated in meniscal regeneration. We identified that LIPUS stimulus enhanced fibrochondrogenic transdifferentiation of VSMCs in vitro and in vivo. Mechanistically, LIPUS stimulus could up-regulate Piezo1 expression, then activate the TGFβ1 signal, following repression of the Notch signal, consequently enhancing fibrochondrogenic transdifferentiation of VSMCs. Finally, the anisotropic native-like meniscal fibrocartilage tissue was acquired by combining PU_PCL scaffold implantation and regular LIPUS stimulus in a beagle canine subtotal meniscectomy model (Fig. 8).

## Ethical Approval

All institutional and national guidelines for the care and use of laboratory animals were followed.

## Data Availability

All data needed to evaluate the conclusions in the paper are present in the paper and/or the Supplementary Materials.
