# Supplementary material for: Vascular Smooth Muscle Cells Transdifferentiate into Chondrocyte-Like Cells and Facilitate Meniscal Fibrocartilage Regeneration
Source: Research (Wash D C). 2024 Dec 23;7:0555. doi: 10.34133/research.0555 (PMC11665451; doi:10.34133/research.0555)
Supplement: Supplementary 1 — Materials and Methods Figs. S1 to S30 Tables S1 to S6 [file research.0555.f1.pdf]

Supplemental information

Vascular smooth muscle cells transdifferentiate into chondrocyte-like cells and facilitate meniscal fibrocartilage regeneration

Wenqiang Yan [1,2,3], Jin Cheng [1,2,3], Haoda Wu [1,2,3], Zeyuan Gao [1,2,3], Zong Li [1,2,3], Chenxi Cao [1,2,3], Qingyang Meng [1,2,3], Yue Wu [1,2,3], Shuang Ren [1,2,3], Fengyuan Zhao [1,2,3], Hongde Wang[1,2,3], Ping Liu[1,2,3*], Jianquan Wang[1,2,3*], Xiaoqing Hu [1,2,3,*], Yingfang Ao [1,2,3,*]

[1] Department of Sports Medicine, Peking University Third Hospital, Institute of Sports Medicine of Peking University, Beijing, China

[2] Beijing Key Laboratory of Sports Injuries, Beijing, China

[3] Engineering Research Center of Sports Trauma Treatment Technology and Devices, Ministry of Education, Beijing, China

[*]**corresponding author:** Yingfang Ao (laimoc@pku.edu.cn), Xiaoqing Hu (huxiaoqingbd01@sina.com) , Jianquan Wang (wjqsportsmed@163.com) and Ping Liu (bjusmlp@126.com)

MATERIALS AND METHODS

Western blot

Total proteins were extracted from rat VSMCs using RIPA lysis buffer (C1053, Applygen, China) containing protease inhibitor (P1265-1, Applygen, China) and phosphatase inhibitor (P1260-1, Applygen, China). Protein concentration was measured by NanoDrop (Thermo Fisher, USA) at 280 nm ultraviolet. The proteins were separated on 4-20% Bis-Tris polyacrylamide gel (M00930, Genscript, China) electrophoresis and then transferred onto polyvinylidene fluoride membranes (PVDF) (ISEQ00010, Millipore, USA) according to standard procedures. The PVDF membranes were blocked with 5% (w/v) bovine serum albumin (BSA) (P1621, Applygen, China) for 1 h at room temperature. The PVDF membranes were incubated with primary antibodies overnight at 4 ℃. After washing with TBST solution, the PVDF membranes were incubated with secondary antibodies for 1 h at room temperature. After thorough washing, the ECL ultra western HRP (horseradish peroxidase) substrate was used to develop the color. Finally, the signals were captured by ChemiDocXRS + Imaging System (Tanon, Shanghai, China).

Quantitative real-time polymerase chain reaction (qPCR)

Total RNA was extracted using TRIzol reagent (15596018, Invitrogen, USA) according to manufacturer's instructions. The concentration of RNA was measured by NanoDrop (Thermo Fisher, USA). The reverse transcription was completed using commercial kit (R323-01, Vazyme, Nanjing, China). The qPCR was performed by magnifying the diluted complementary DNA of 20 μL with SYBR Green Q-PCR Kit

(Q141-03, Vazyme, Nanjing, China) using the Applied Biosystems StepOnePlus Real-Time PCR System (Foster City, CA, USA). The gene expression was calculated using the $2^{-\Delta\Delta CT}$ and represented with fold-change. The primer sequences of rat VSMCs were demonstrated in **Supplemental table 6**.

Tissue immunofluorescence, immunohistochemistry, two-photon microscopy, semiquantitative analysis

For tissue immunofluorescence, firstly, 3 μm-thick paraffin sections were prepared using microtome (Leica, Germany). The sections were immersed into xylene and graded ethanol to deparaffinize and regain water. The heat induced antigen retrieval was completed using pH 6.0 citric acid for 20 min. The nonspecific protein binding was blocked using goat serum (Boster, AR0009, China) for 1 hour at room temperature. The sections were incubated with corresponding primary antibodies for 2 h at room temperature. After thorough washing with PBST, the sections were incubated with corresponding secondary antibodies for 1 hour at room temperature, followed by DAPI incubation. Finally, the slices were sealed with anti-fluorescence quenching agent. The confocal microscope (Leica, Germany) was sued to capture images. For semiquantitative analysis, the integrated intensity of corresponding target in region of interest (ROI) was evaluated by Image J software (US National Institutes of Health, USA).

For tissue immunohistochemistry, the procedures before secondary antibody incubation were identical to those of tissue immunofluorescence. The corresponding secondary antibodies containing HRP were used. After thorough washing with PBST,

the color was developed using diaminobenzidine (DAB) substrate kit. Finally, the slices were scanned by a digital scanner (NanoZoomer, Hamamatsu, Japan).

For collagen assessment within regenerated tissue, the sections were firstly immersed into xylene and graded ethanol to deparaffinize and regain water. The two-photon microscopy (TCS-SP8 DIVE, Leica, Germany) was used to acquire the second harmonic generation (SHG) signal associated with collagen matrix. The SHG signal was acquired with 850 nm excitation and 415-435 nm emission wavelength.

Co-staining of SOX9 immunofluorescence and Biotin-labeled in Situ Hybridization (ISH) for SRY gene

The probe for SRY gene was designed according to a previous study [23]. The probe sequence was as follows: SRY probe (5’ and 3’ biotin) TGCAAGCAGCAAACTGTCGCT (Qiagen, Germany). All solutions used for ISH were free of ribonuclease. The paraffin-embedded sections were immersed into xylene and ethanol to deparaffinize and regain water. The sections were permeabilized by 20 ug/ml proteinase K solution at 37 °C for 15 minutes and then washed with PBS. The heat induced antigen retrieval was completed using pH 6.0 citric acid for 20 min. Then, the slices were treated with prehybridization buffer for 1 hour at 37 °C. The tissue section was incubated with hybridization mix (hybridization buffer with SRY probe, 80 nM) for 1 hour at 54 °C. After hybridization, rigorous wash was completed with graded sodium citrate buffer (SSC) and PBS. Nonspecific protein binding was blocked by 1% bovine serum albumin (BSA) at room temperature for 15 minutes. Then, the slices were incubated with FITC-labeled anti-biotin and SOX9 antibody for 1 hour at room

temperature. Then, the slices were incubated with secondary antibody and DAPI for 30 minutes. After thorough washing with PBST, the slices were sealed with anti-fluorescence quenching agent. The confocal microscope (TCS-SP8, Leica) was used to capture the images.

Nanoindentation test

The nanoindentation test was completed according to a previous study [25]. Firstly, the sample was glued to a glass slide with surface perpendicular to the indentation direction. The rubber ring was then glued to the glass slide to enclose the sample tissue. The PBS solution was added to the rubber ring to keep the samples hydrated. Taking care to avoid sample drying or dehydration during the entire process. A Tribo-Indenter (Hysitron) with a 400 μm radius curvature, conospherical, diamond probe tip was used. Random six points through each sample were measured. The trapezoidal load function with load (10 seconds), hold (2 seconds), and unload (10 seconds) was performed. The displacement control model with maximal 500 nm depth was used. Finally, the elastic modulus and hardness were calculated.

RNA sequencing (RNA-seq) of rat VSMCs

The RNA-seq analyses of rat VSMCs after LIPUS treatment were performed using the Dr.TOM Platform (https://biosys.bgi.com). Total RNA was extracted using TRIzol reagent. cDNA libraries were constructed for each pooled RNA sample using MGIEasy RNA Directional kit Total RNA-seq. Bowtie2(v2.2.5) was used to align the clean reads to the gene set. The gene expression level was calculated by RSEM (v1.3.1). The gene expression was determined by TPM method. The differentially expressed genes were

identified by DESeq2(v1.4.5) algorithm. Significant analysis was completed using the *P*-value and false discovery rate (FDR) analyses. The genes with a fold change >2 or fold change <0.5, FDR < 0.05 were considered to be differentially expressed. To take insight to the change of phenotype, GO (http://www.geneontology.org/) and KEGG (https://www.kegg.jp/) enrichment analysis of annotated different expression gene was performed by Phyper (https://en.wikipedia.org/wiki/Hypergeometric_distribution) based on Hypergeometric test. The significantly affected GO categories and pathways were identified by Fisher's exact test. The *P* value was used to define the threshold of significance.

Single cell RNA-sequencing (scRNA-Seq) of regenerated and native beagle canine meniscal tissue

The enzyme solution containing 2% type I collagenase and 2% type II collagenase was prepared using centrifuge tubes and preheated into 37°C in water bath. The meniscal tissue was cut into small pieces (1*1*1 mm) using scalpel. The tissue block was transferred into a centrifuge tube containing enzymatic hydrolysate and placed in the constant temperature shaker at 37 °C for digestion. The enzymatic solution was shaken gently. The tissue digestion was examined via trypan blue staining until no obvious tissue block existed, then the digestion could be paused. The cell suspension was filtered using 40μm cell strainer. The residual cells were cleaned using 2-3mL precooled complete medium in which the cleaning solution was filtered and collected in a centrifuge tube. Centrifuge at 300g and 4°C for 5 mins and discard the supernatant. Cell status was observed via trypan blue staining. The dead cells and debris were

removed using Dead Cell Removal Kit. The PBS-BSA (0.04%) was used to wash cells. Centrifuge at 300g and 4℃ for 5 mins and discard the supernatant. An appropriate amount of PBS-BSA (0.04%) was used to resuspend the precipitation followed by counting the cells using a hemacytometer.

The single-cell suspension, oil, beads were added into C4 scRNA slide and instrument in order. Cell lysis and magnetic bead capture mRNA were performed in droplets. Prepare reagents for emulsion breakage in advance, vacuum pump was used for emulsion breakage. The reaction system was configured. After reacting at the suitable temperature for a fixed period of time, reverse transcription was conducted. Then, cDNA second strand was synthesized after reacting at the suitable temperature for a fixed period of time. After reacting at the suitable temperature for a fixed period of time, cDNA and oligo products were amplified. The purification process was performed separately. The cDNA and oligo products were subjected to quality detection of their concentration and the size distribution of the fragments. Afterwards, Oligo products were subjected to amplification, index ligation and purification for further circularization. The cDNAs were subjected to fragmentation, end repair and addition of “A” base at the 3'-end of each strand followed by purification. The cDNAs were subjected to adaptor ligation followed by purification. The PCR reaction system was configured. After reacting at the suitable temperature for a fixed period of time, amplification was processed via PCR followed by purification. The corresponding library quality control was completed. cDNA and Oligo products were respectively denatured into single strand. The reaction system and program for circularization were

respectively configured and set up. Single-stranded cyclized products were produced, while uncyclized linear DNA molecules were digested. Single-stranded circle DNA molecules were replicated via rolling cycle amplification, and a DNA nanoball (DNB) which contain multiple copies of DNA was generated. Sufficient quality DNBs were then loaded into patterned nanoarrays using high-intensity DNA nanochip technique and sequenced through combinatorial Probe-Anchor Synthesis (cPAS).

The raw gene expression matrix generated by processing raw sequencing data for each sample using DNBelab_C4scRNA (v1.0.1) [28]. Downstream analysis was completed using the R package Seurat (v 3.2.0) [29]. The quality control was performed based on the number of detected genes and proportion of mitochondrial reads per cell. Specifically, cells with less than 200 detected genes or cells with >90% of the maximum genes were filtered out. For the mitochondrial metric, cells were sorted in descending order according to the mitochondrial read ratio, the top 15% of cells were filtered out. Potential doublets were identified and removed by DoubletDetection (https://rdrr.io/github/scfurl/m3addon/man/doubletdetection.html). The Cell cycle analysis was performed using the CellCycleScoring function in Seurat program. The gene expression dataset was normalized followed by principal component analysis (n=15) using the 2000 highly variable genes in the dataset. U-MAP was then used for two-dimensional visualization of the resulting clusters. For each cluster, the marker genes were identified using the FindAllMarkers function as implemented in the Seurat package (logfc. threshold>0.25, minPct>0.1 and Padj≤0.05). Then, clusters were remarked to known cell types by SCSA method [30]. Differentially expressed gene

across different samples were identified using the FindMarkers function in Seurat with parameters 'logfc. threshold>0.25, minPct>0.1 and Padj≤0.05'.

GO (associated three integrated databases: Uniprot: http://ftp.ebi.ac.uk/pub/databases/GO/goa/UNIPROT/goa_uniprot_all.gaf.gz, NCBI's gene2GO ftp://ftp.ncbi.nih.gov/gene/DATA/gene2go.gz, GO's official website: ftp://ftp.pir.georgetown.edu/databases/idmapping/idmapping.tb.gz) analysis and KEGG (V93.0) pathway analysis were performed using phyper, a function of R. Only GO terms or KEGG pathways with FDR<=0.05 were considered to be significantly enriched. The Monocle2 [31] applied reversed graph embedding to describe multiple fate decisions in a fully unsupervised manner. We used Monocle2 to do Pseudo-time analysis.

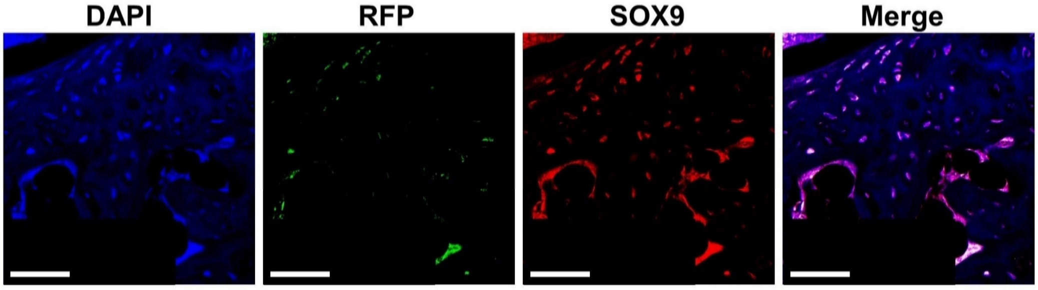

Supplemental Figure 1. The immunofluorescent co-staining of RFP and SOX9 in the healthy native meniscus of *Myh11-CreER^{T2}; Rosa26-LSL-Tdtomato* smooth muscle lineage tracing transgenic mice. RFP, red fluorescence protein. RFP signals represent Myh11 positive cells. Scale bar: 50 µm.

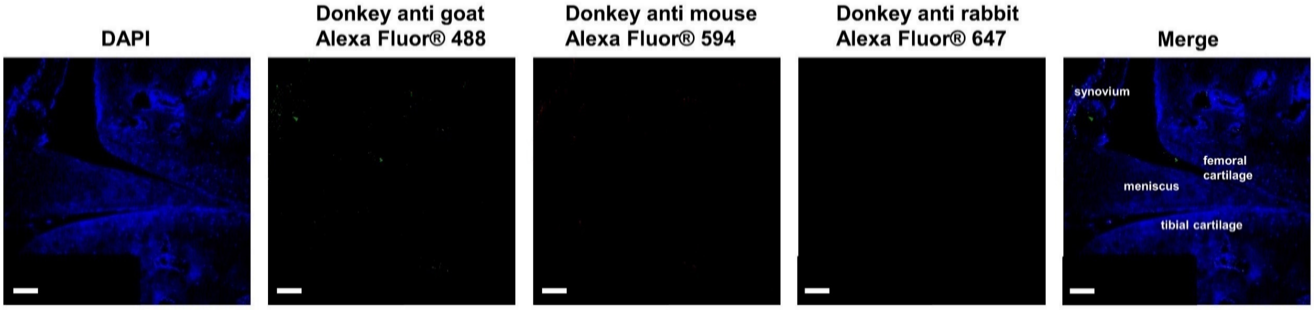

Supplemental Figure 2. The negative control of healthy native mice knee immunofluorescence. Scale bar: 50 µm.

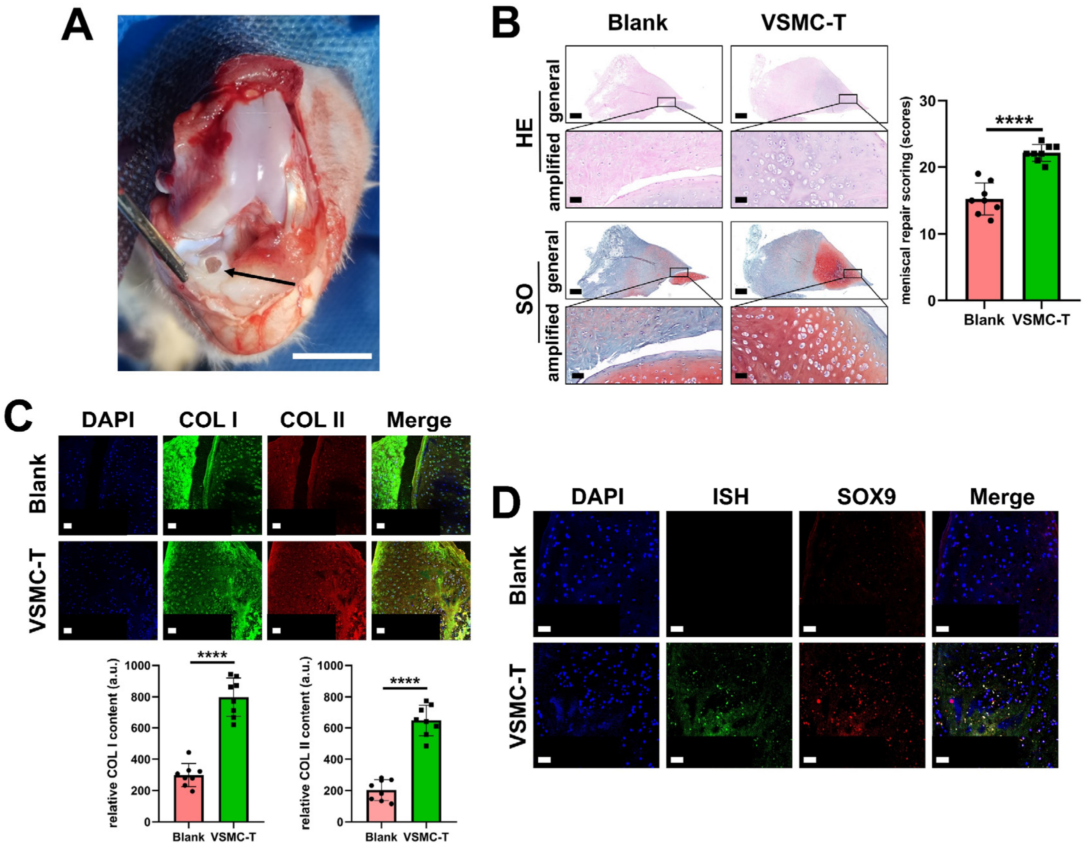

Supplemental Figure 3. VSMCs transplantation facilitates meniscal repair in a rabbit meniscus focal defect model. (A) The preparation of rabbit meniscal focal defect model and VSMCs transplantation. The VSMCs are encapsulated in the Gel-MA hydrogel and implanted into the defect as indicated by the black arrow. scale bar: 1 cm. A total of eight adult rabbits (female, 6 months) were included. Four rabbits (eight knees) were used as VSMCs transplant group. Another four rabbits (eight knees) were used as blank group. The cylindrical full-thickness defects with a diameter of 2.0 mm were prepared in the avascular portion of anterior horn of medial menisci in bilateral knees. The samples were harvested for histological analyses at 12 weeks post surgery. **(B)** The histological analysis of repaired meniscal tissue and meniscal repair scoring in Blank and VSMC-T group, HE represents hematoxylin-eosin, SO represents safranin O-fast green staining, VSMC-T represents VSMC transplantation, scale bar of general: 0.5

mm, scale bar of amplified: 0.05 mm; n=8, unpaired *t*-test, **** represents p<0.001. **(C)** The immunofluorescent co-staining of COL I and COL II in repaired meniscal tissue and semiquantitative analysis of COL I and COL II. scale bar: 50 μm, n=8, unpaired *t*-test, **** represents p<0.001. **(D)** The transplanted VSMCs transdifferentiates into chondrocyte-like cells confirmed by co-staining of ISH for SRY gene and SOX9 immunofluorescence, scale bar: 50 μm.

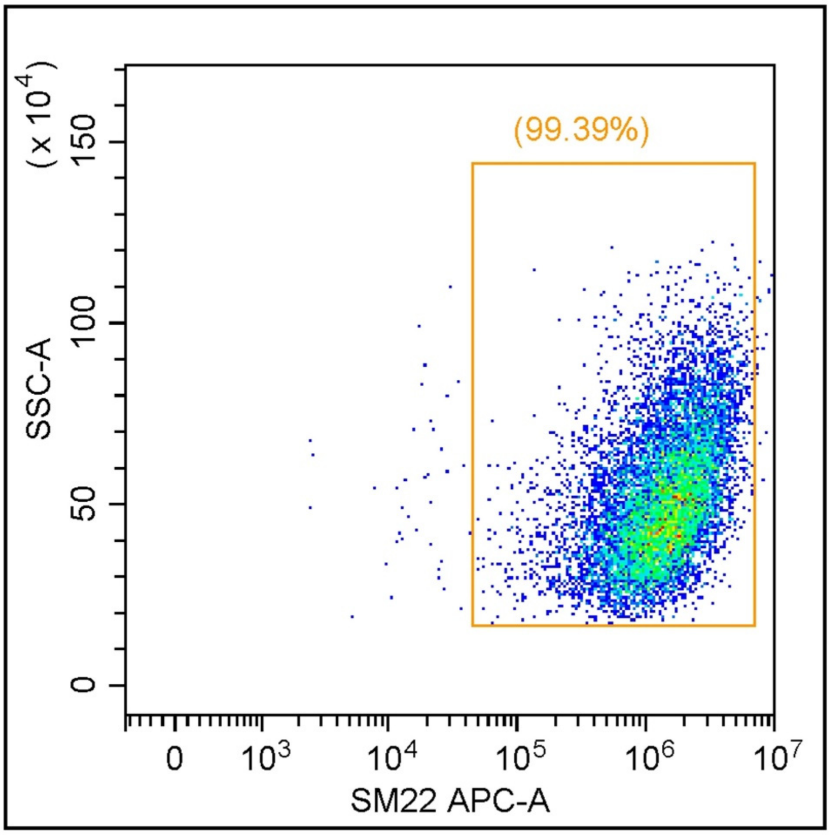

Supplemental Figure 4. The flow cytometry analysis of isolated rat vascular smooth muscle cells based on SM22alpha marker.

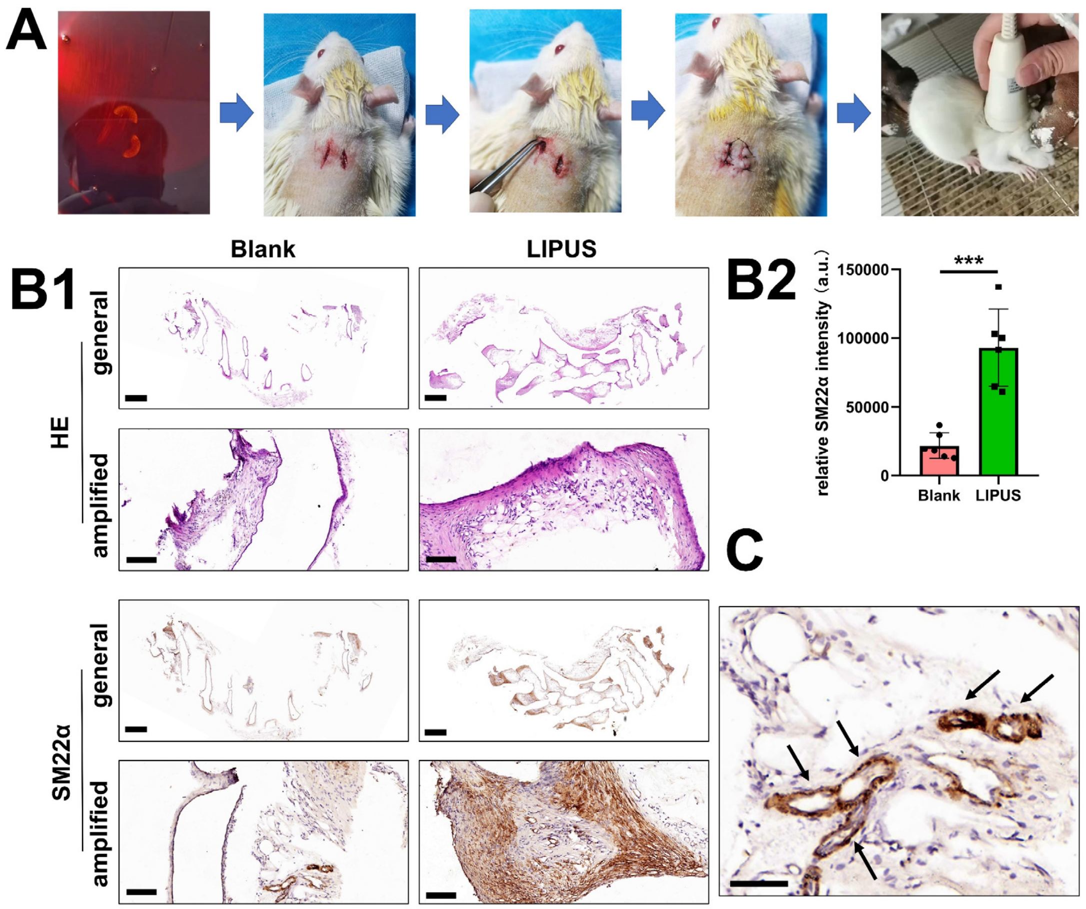

Supplemental Figure 5. LIPUS facilitates VSMCs migration into porous scaffold in a rat model. (A) The subcutaneous implantation of porous scaffold in the back of rat, followed by LIPUS stimulus. 6 rats (male, 2 months) were used. The LIPUS stimulus group and non-LIPUS blank group were included. Each group contained 3 rats (6 samples). All samples were harvested for histological analysis at 12 weeks post surgery. **(B)** The histological and immunohistochemical analyses of VSMC migration into the scaffold (**B1,** the HE staining and SM22alpha immunohistochemical staining, scale bar of general: 1 mm, scale bar of amplified: 0.1 mm; **B2,** semiquantitative analysis of SM22alpha intensity, n=6, unpaired *t*-test). *** represents $p<0.005$. **(C)** The validation of using SM22alpha as VSMC marker. As shown by the amplified

immunohistochemical staining of Blank group, SM22alpha signal was restricted in the blood vessels. The black arrows indicate blood vessels. Scale bar: 0.05 mm.

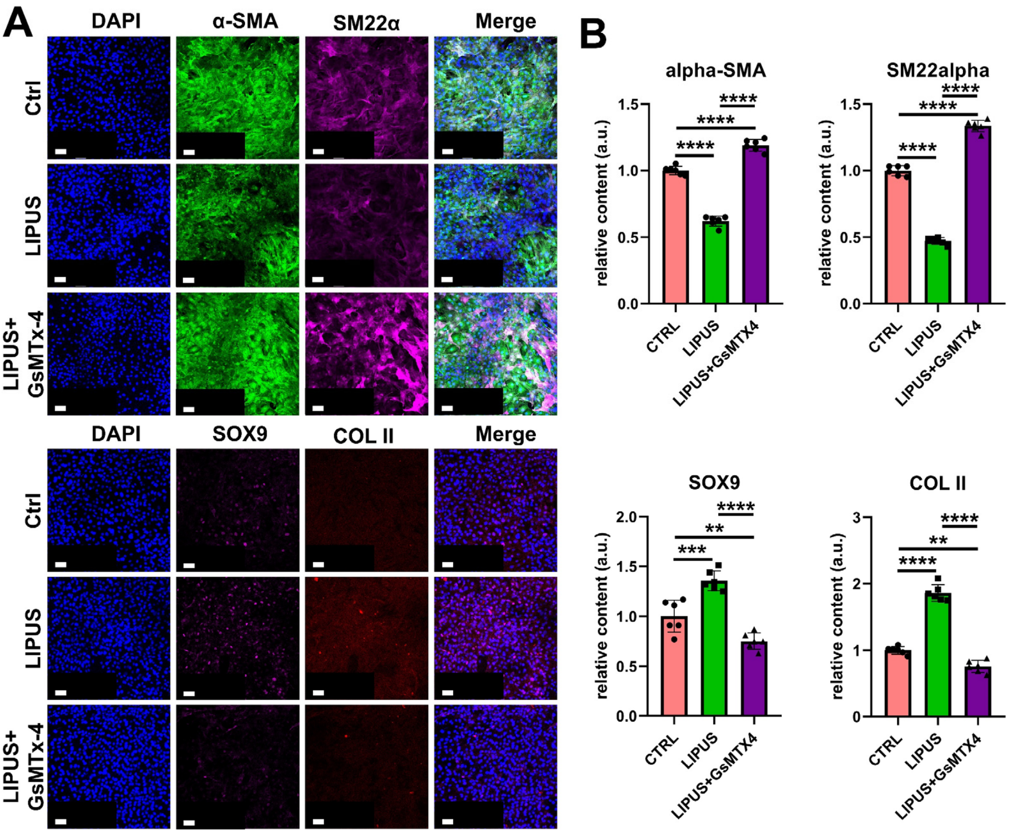

Supplemental Figure 6. GsMTx-4 treatment blocks the effect of LIPUS on rat VSMCs chondrogenic transdifferentiation. **(A)** the immunofluorescence analysis on alpha-SMA, SM22alpha, SOX9 and COL II, scale bar: 50 μm. **(B)** the semiquantitative analysis on the relative expression of alpha-SMA, SM22alpha, SOX9 and COL II. n=6, one-way ANOVA, ** represents $p<0.01$, *** represents $p<0.005$, **** represents $p<0.001$.

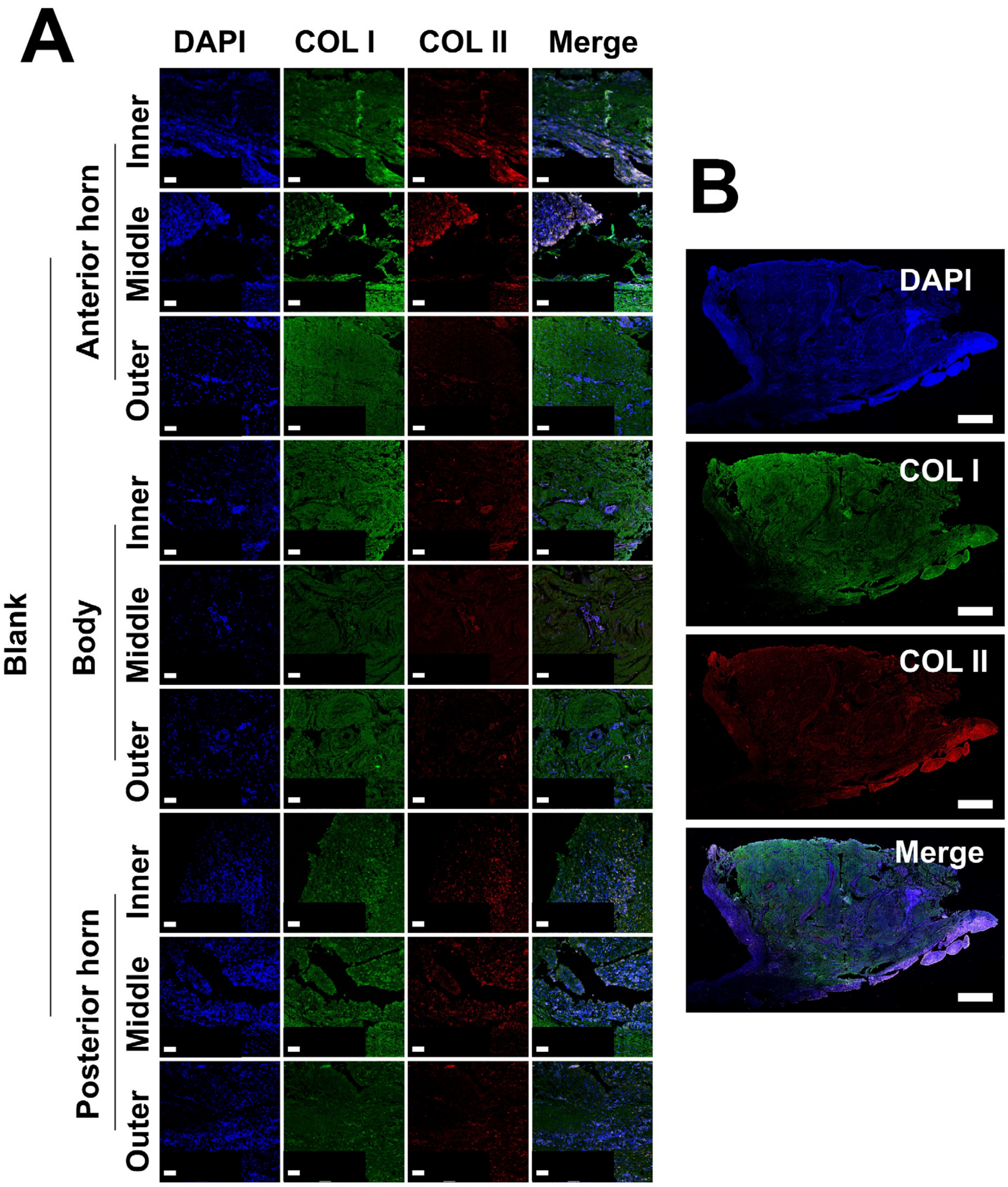

Supplemental Figure 7. The immunofluorescent assessment of COL I and COL II in regenerated tissue of beagle canine blank group. (A) The representative amplified immunofluorescent images in subregions. Scale bar: 50 μm. **(B)** The general image of whole tissue section. Scale bar: 1 mm.

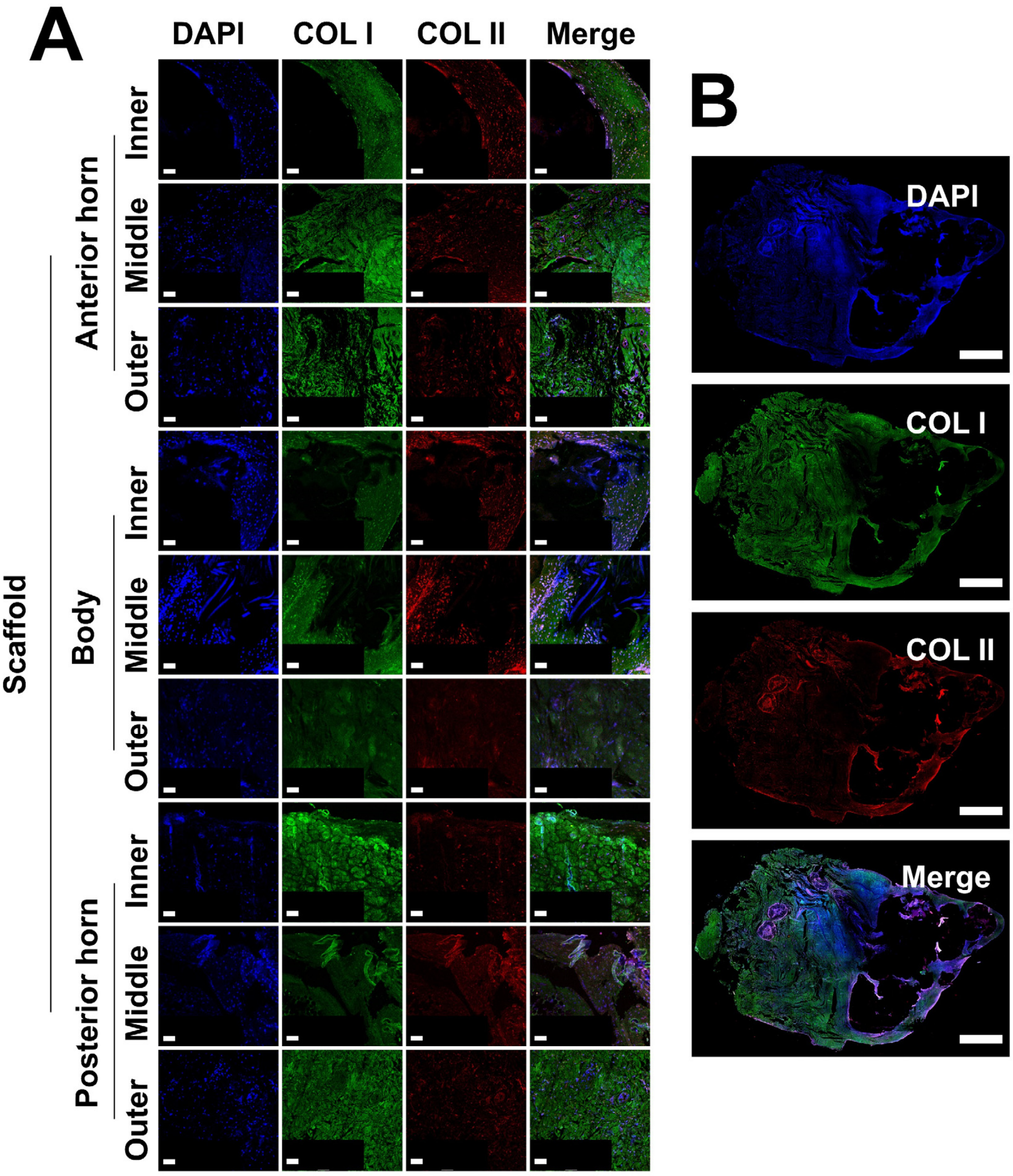

Supplemental Figure 8. The immunofluorescent assessment of COL I and COL II in regenerated tissue of beagle canine Scaffold group. (A) The representative amplified immunofluorescent images in subregions. Scale bar: 50 μm. **(B)** The general image of whole tissue section. Scale bar: 1 mm.

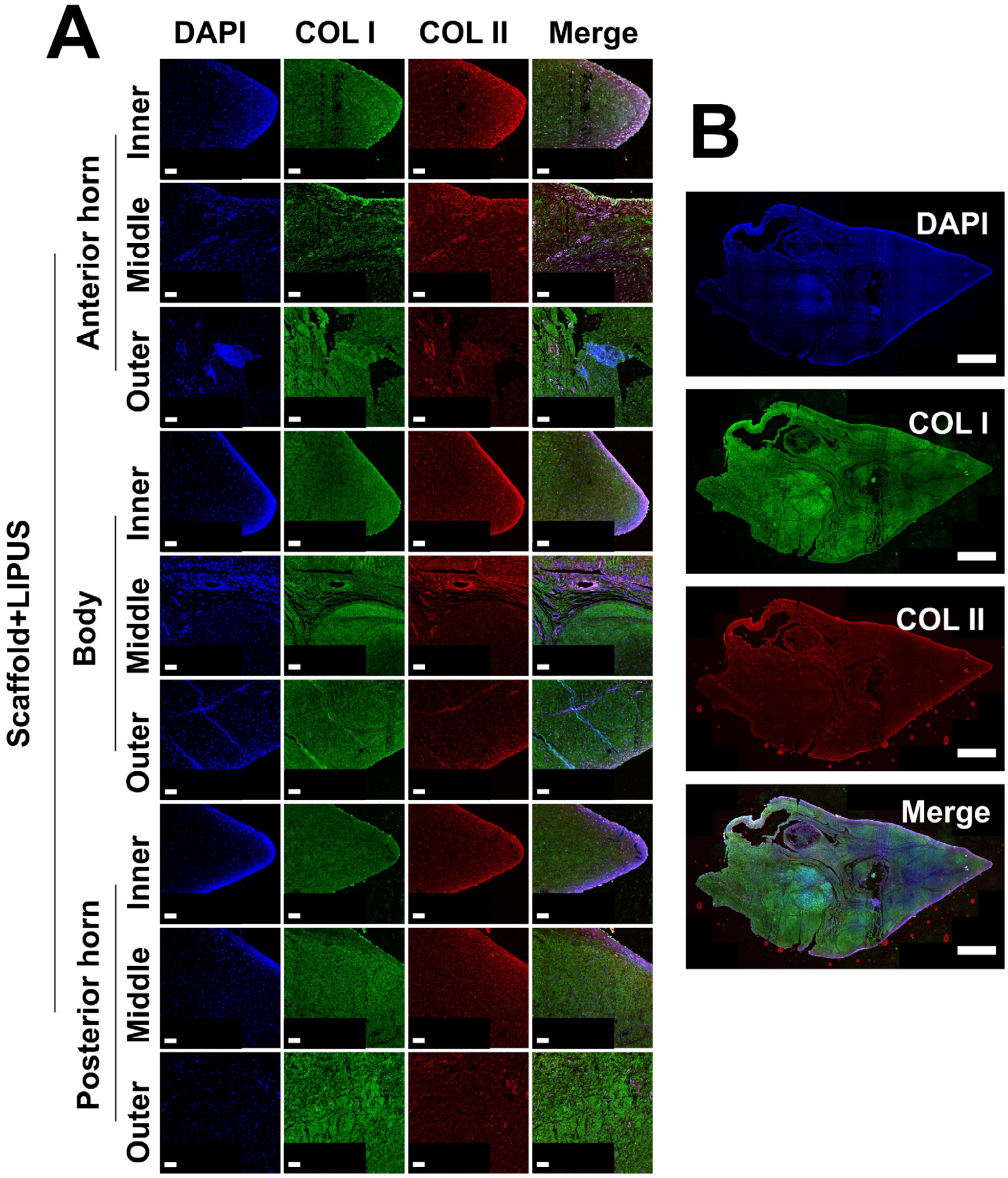

Supplemental Figure 9. The immunofluorescent assessment of COL I and COL II in regenerated tissue of beagle canine Scaffold+LIPUS group. (A) The representative amplified immunofluorescent images in subregions. Scale bar: 50 µm. **(B)** The general image of whole tissue section. Scale bar: 1 mm.

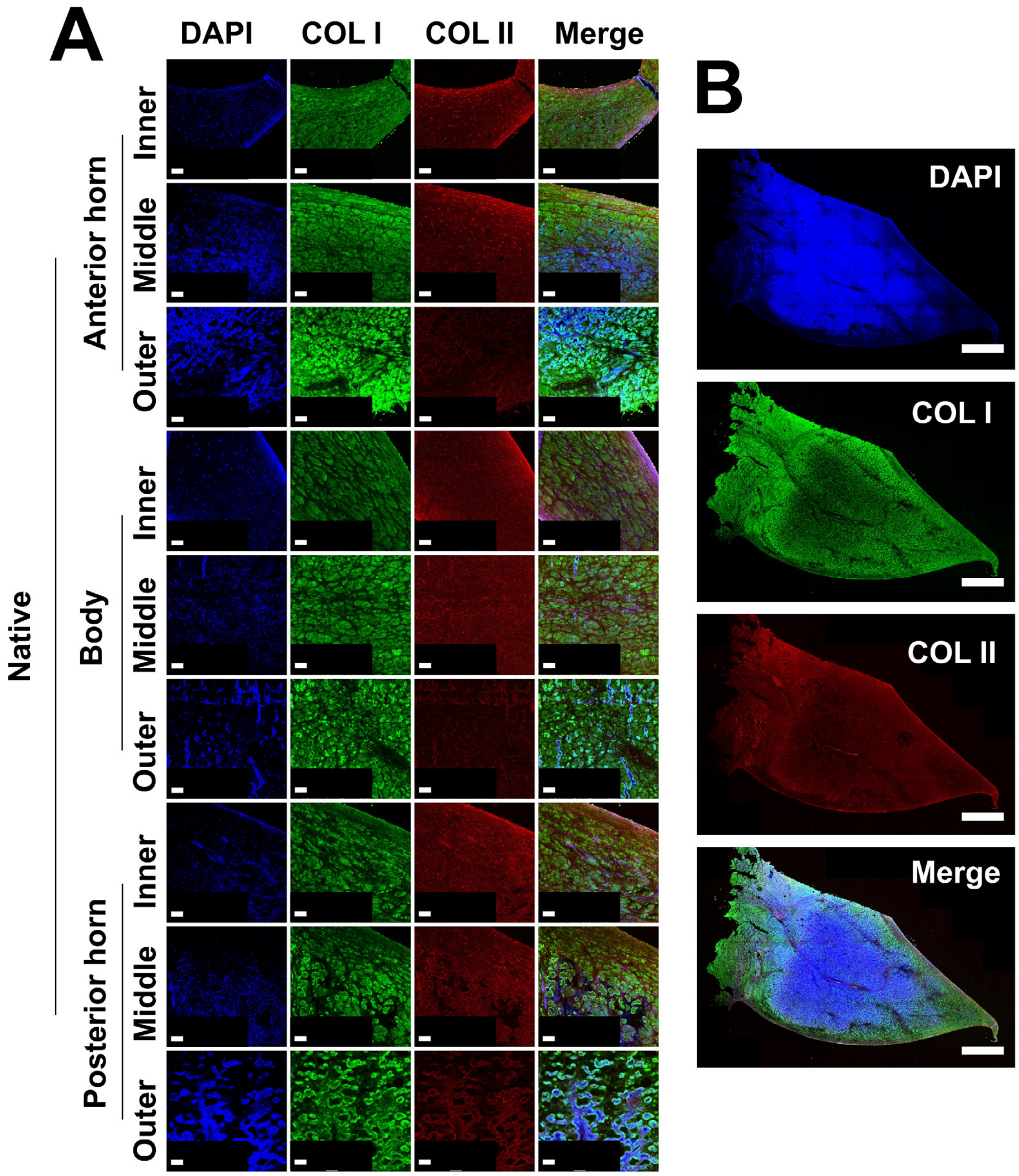

Supplemental Figure 10. The immunofluorescent assessment of COL I and COL II in regenerated tissue of beagle canine Native group. (A) The representative amplified immunofluorescent images in subregions. Scale bar: 50 μm. **(B)** The general image of whole tissue section. Scale bar: 1 mm.

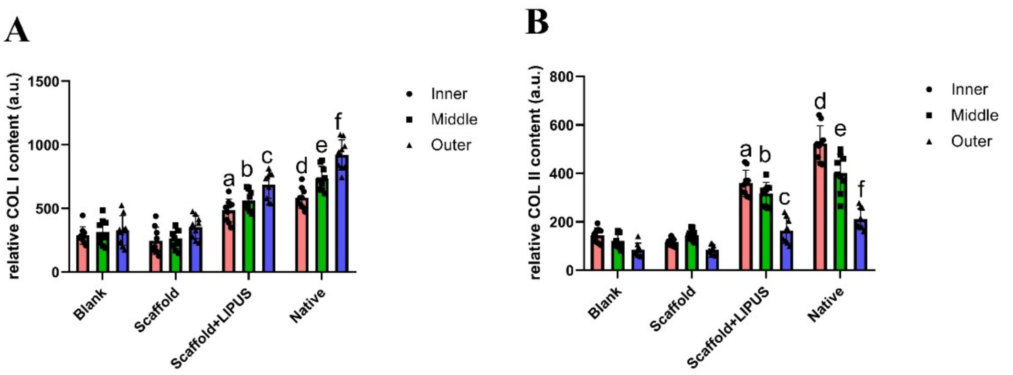

Supplemental Figure 11. The semiquantitative analysis of COL I and COL II in regenerated tissue and native meniscus. (A) the semiquantitative analysis of COL I, n=9, two-way ANOVA, a: $p<0.05$ compared to inner of Blank, Scaffold, Native group; and middle, outer of Scaffold+LIPUS group; b: $p<0.05$ compared to middle of Blank, Scaffold, Native group; and outer of Scaffold+LIPUS group; c: $p<0.05$ compared to outer of Blank, Scaffold, Native group; d: $p<0.05$ compared to inner of Blank, Scaffold group; and outer of Native group; e: $p<0.05$ compared to middle of Blank, Scaffold group; and outer of Native group; f: $p<0.05$ compared to outer of Blank, Scaffold group. **(B)** the semiquantitative analysis of COL II, n=9, two-way ANOVA, a: $p<0.05$ compared to inner of Blank, Scaffold, Native group; and outer of Scaffold+LIPUS group; b: $p<0.05$ compared to middle of Blank, Scaffold, Native group; and outer of Scaffold+LIPUS group; c: $p<0.05$ compared to outer of Blank, Scaffold group; d: $p<0.05$ compared to inner of Blank, Scaffold group; and Middle, outer of Native group; e: $p<0.05$ compared to middle of Blank, Scaffold group; and outer of Native group; f: $p<0.05$ compared to outer of Blank, Scaffold group.

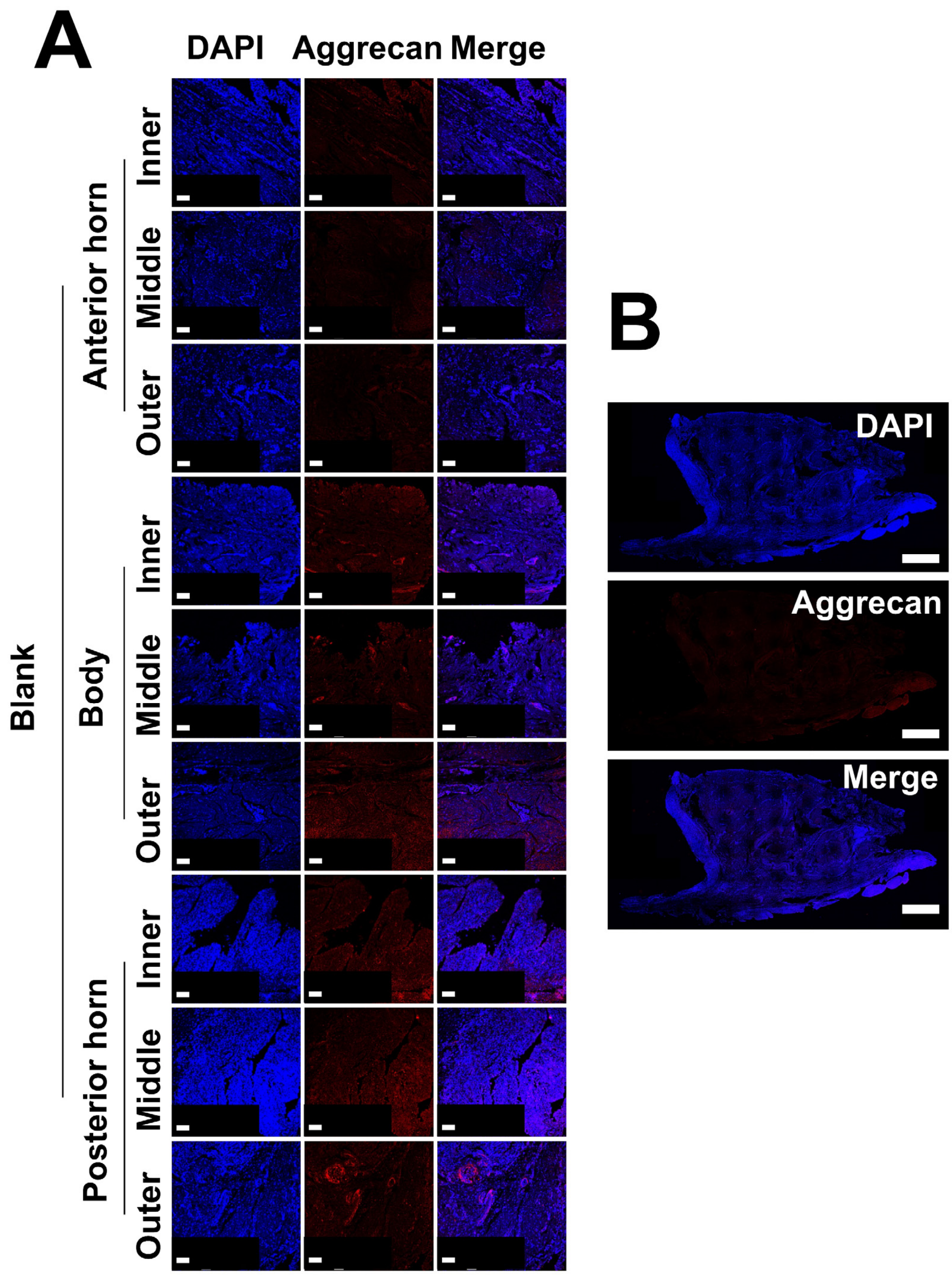

Supplemental Figure 12. The immunofluorescent assessment of Aggrecan in regenerated tissue of beagle canine blank group. (A) The representative amplified immunofluorescent images in subregions. Scale bar: 50 μm. **(B)** The general image of whole tissue section. Scale bar: 1 mm.

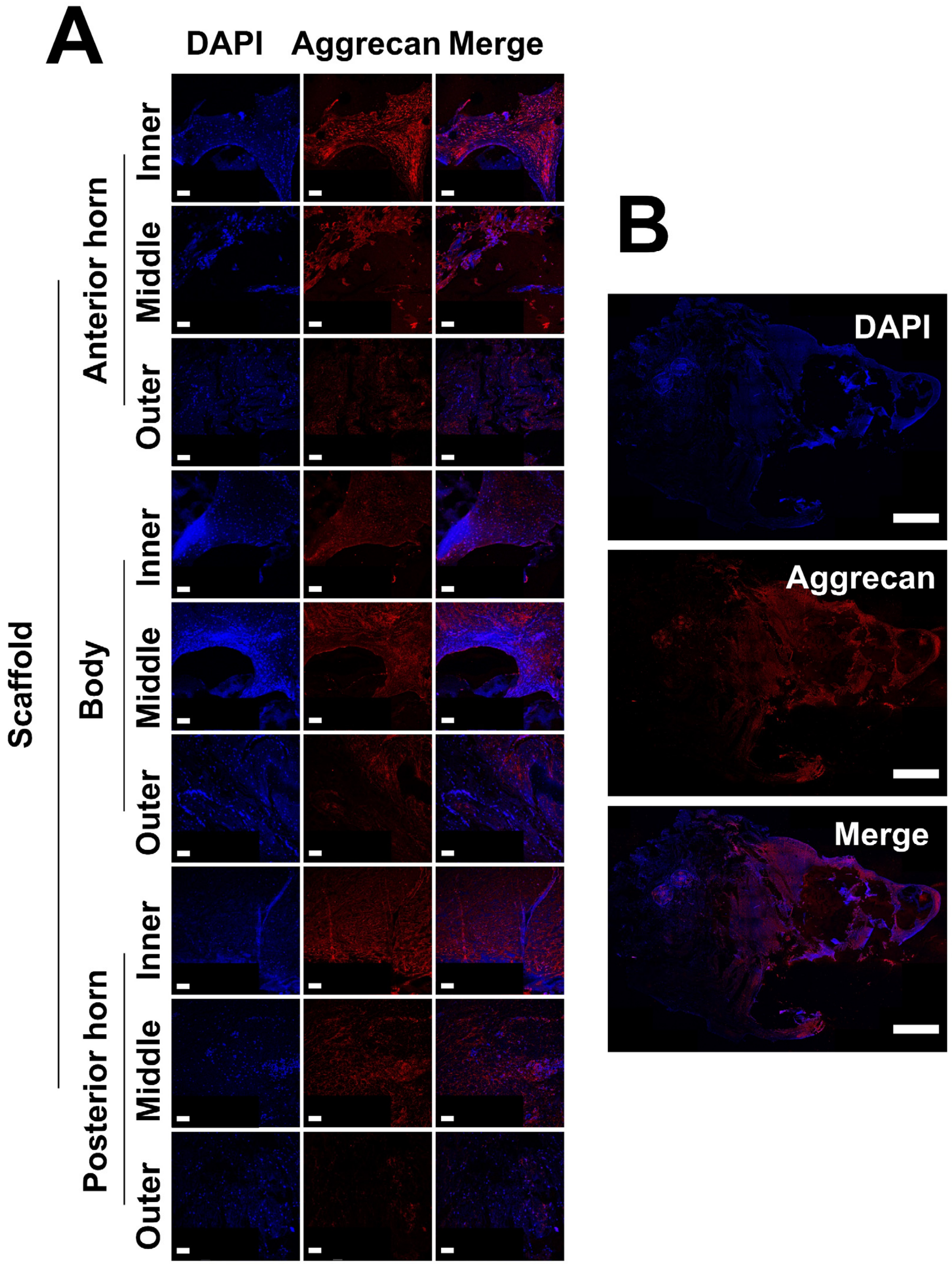

Supplemental Figure 13. The immunofluorescent assessment of Aggrecan in regenerated tissue of beagle canine Scaffold group. (A) The representative amplified immunofluorescent images in subregions. Scale bar: 50 μm. **(B)** The general image of whole tissue section. Scale bar: 1 mm.

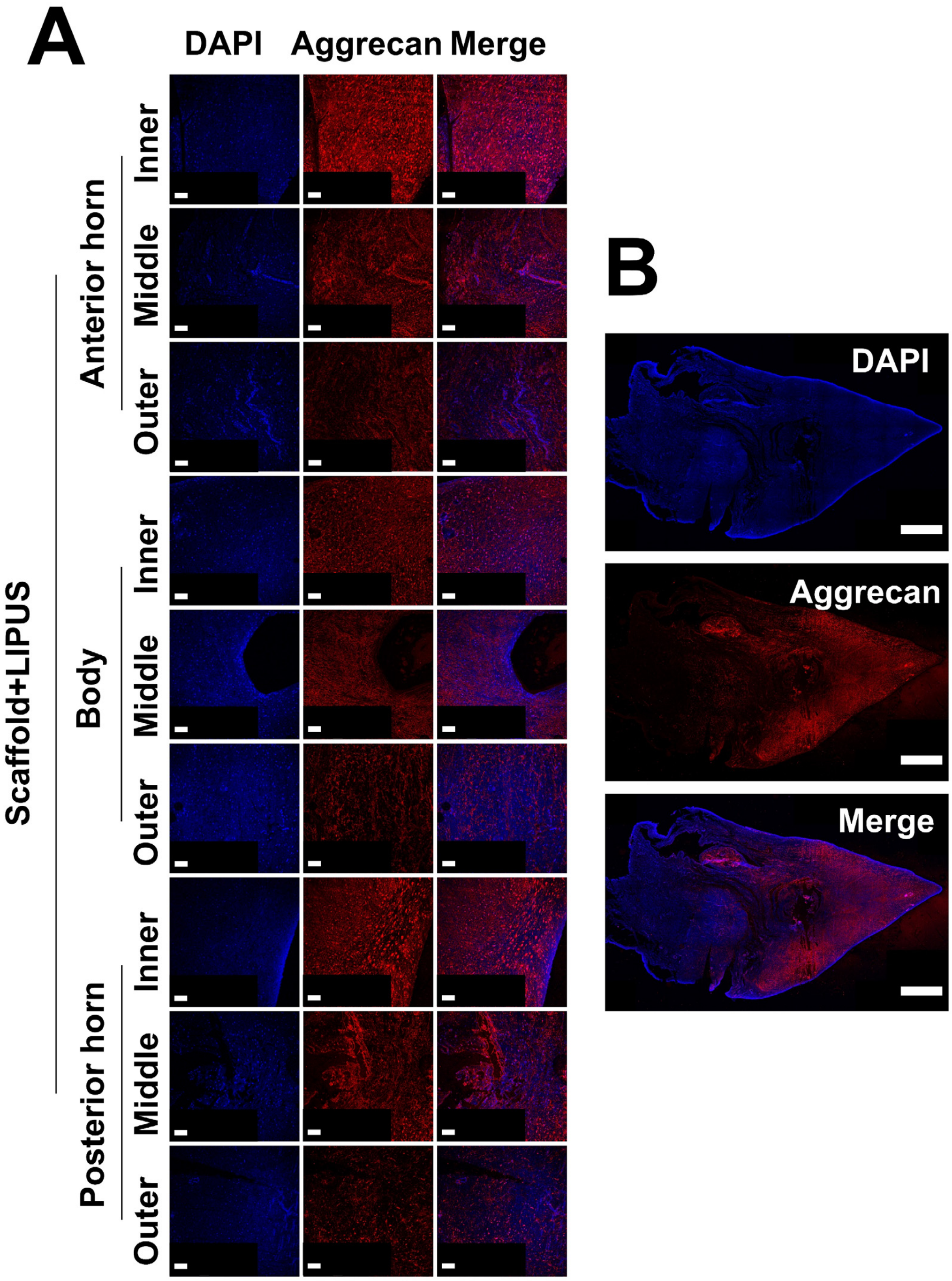

Supplemental Figure 14. The immunofluorescent assessment of Aggrecan in regenerated tissue of beagle canine Scaffold+LIPUS group. (A) The representative amplified immunofluorescent images in subregions. Scale bar: 50 μm. **(B)** The general image of whole tissue section. Scale bar: 1 mm.

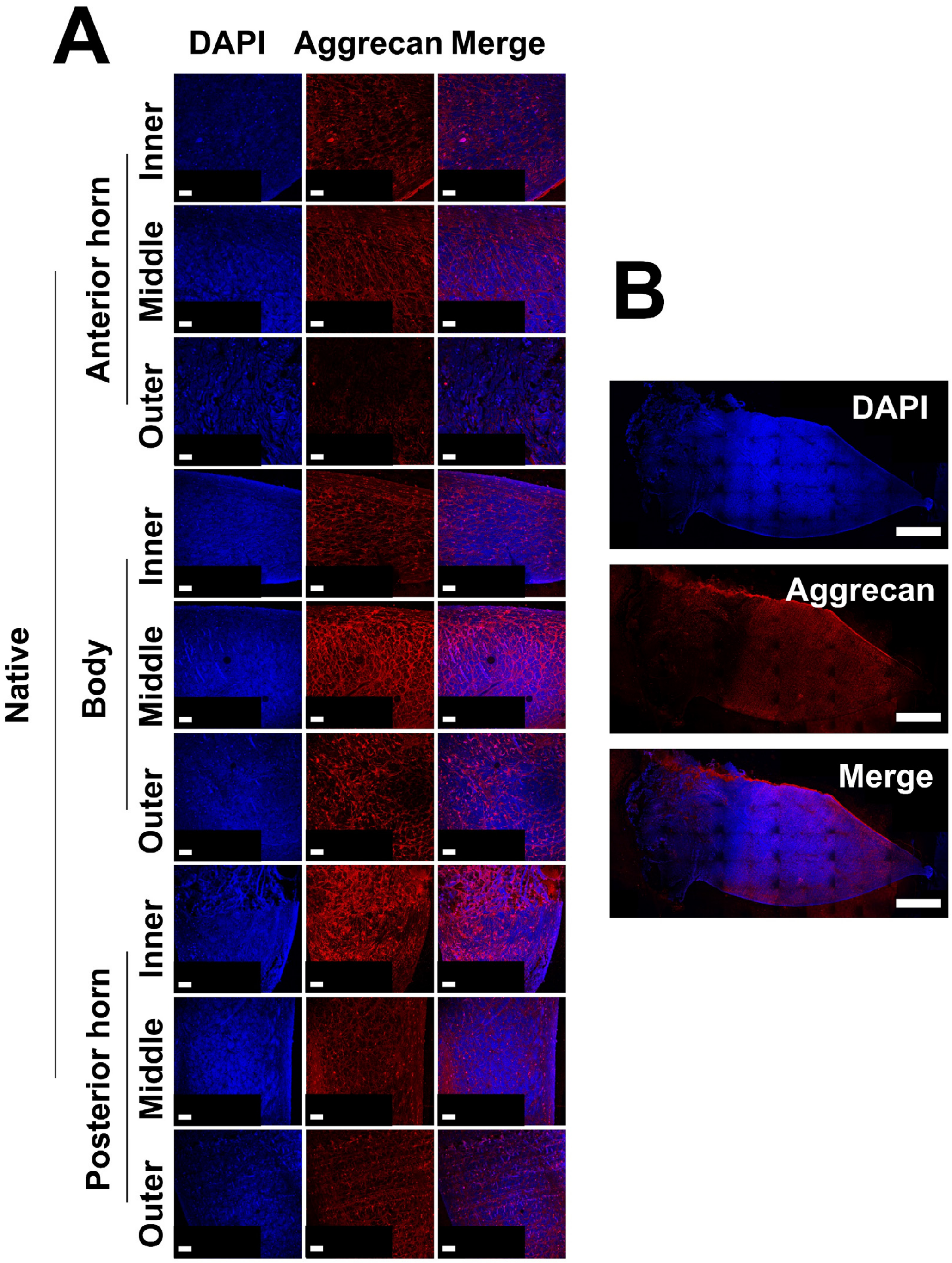

Supplemental Figure 15. The immunofluorescent assessment of Aggrecan in regenerated tissue of beagle canine Native group. (A) The representative amplified immunofluorescent images in subregions. Scale bar: 50 μm. **(B)** The general image of whole tissue section. Scale bar: 1 mm.

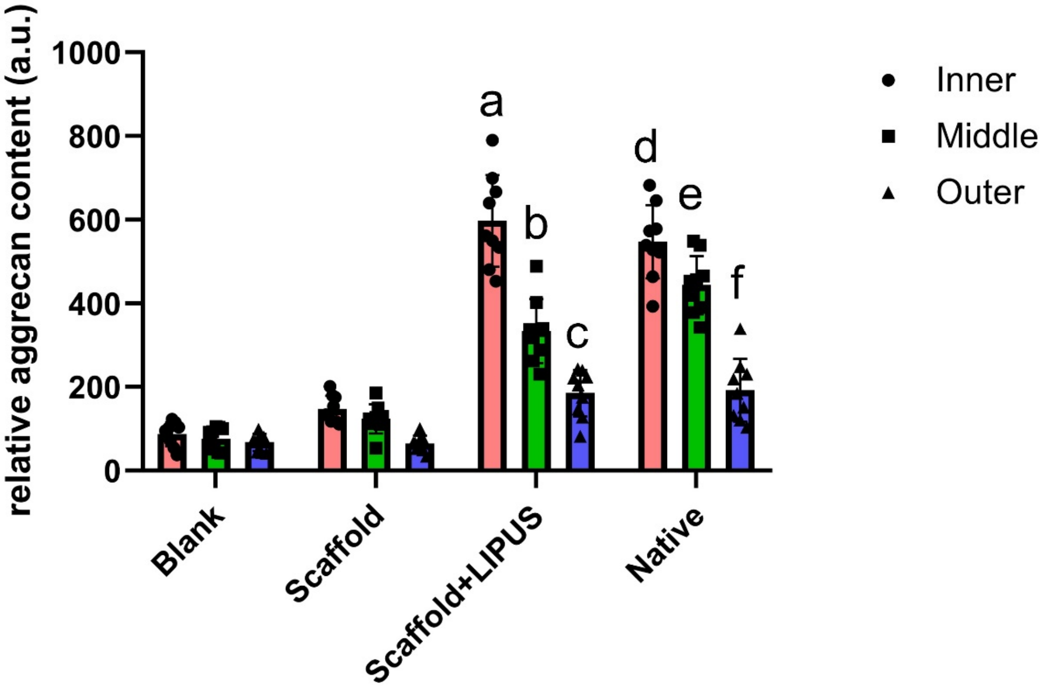

Supplemental Figure 16. The semiquantitative analysis of aggrecan in regenerated tissue and native meniscus, n=9, two-way ANOVA, a: p<0.05 compared to inner of Blank, Scaffold, Native group; and middle, outer of Scaffold+LIPUS group; b: p<0.05 compared to middle of Blank, Scaffold, Native group; and outer of Scaffold+LIPUS group; c: p<0.05 compared to outer of Blank, Scaffold group; d: p<0.05 compared to inner of Blank, Scaffold group; and Middle, outer of Native group; e: p<0.05 compared to middle of Blank, Scaffold group; and outer of Native group; f: p<0.05 compared to outer of Blank, Scaffold group.

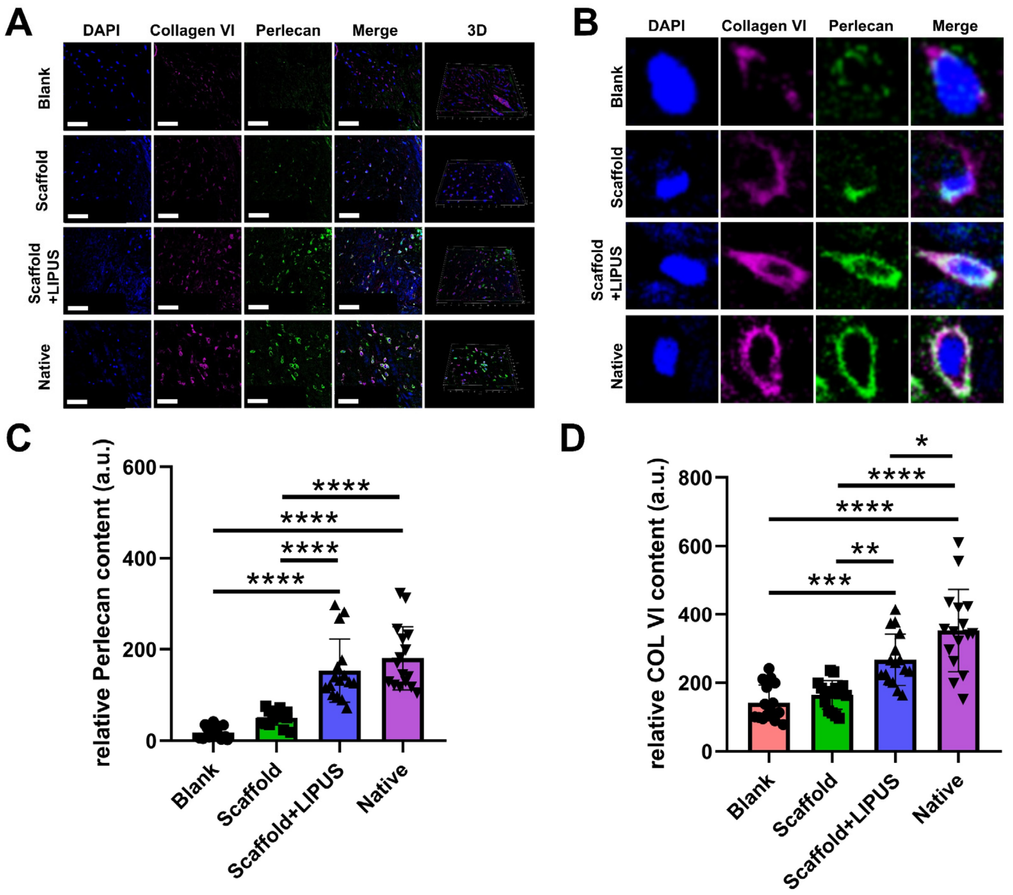

Supplemental Figure 17. The immunofluorescent analysis of pericellular matrix in regenerated tissue and native menisci of beagle canine. (A) the immunofluorescent co-staining of perlecan and COL VI. Scale bar: 50 µm. **(B)** the representative amplified immunofluorescent images. **(C)** the semiquantitative analysis of perlecan content, n=16, one-way ANOVA. **(D)** the semiquantitative analysis of COL VI content, n=16, one-way ANOVA. * represents p<0.05, ** represents p<0.01, *** represents p<0.005, **** represents p<0.001.

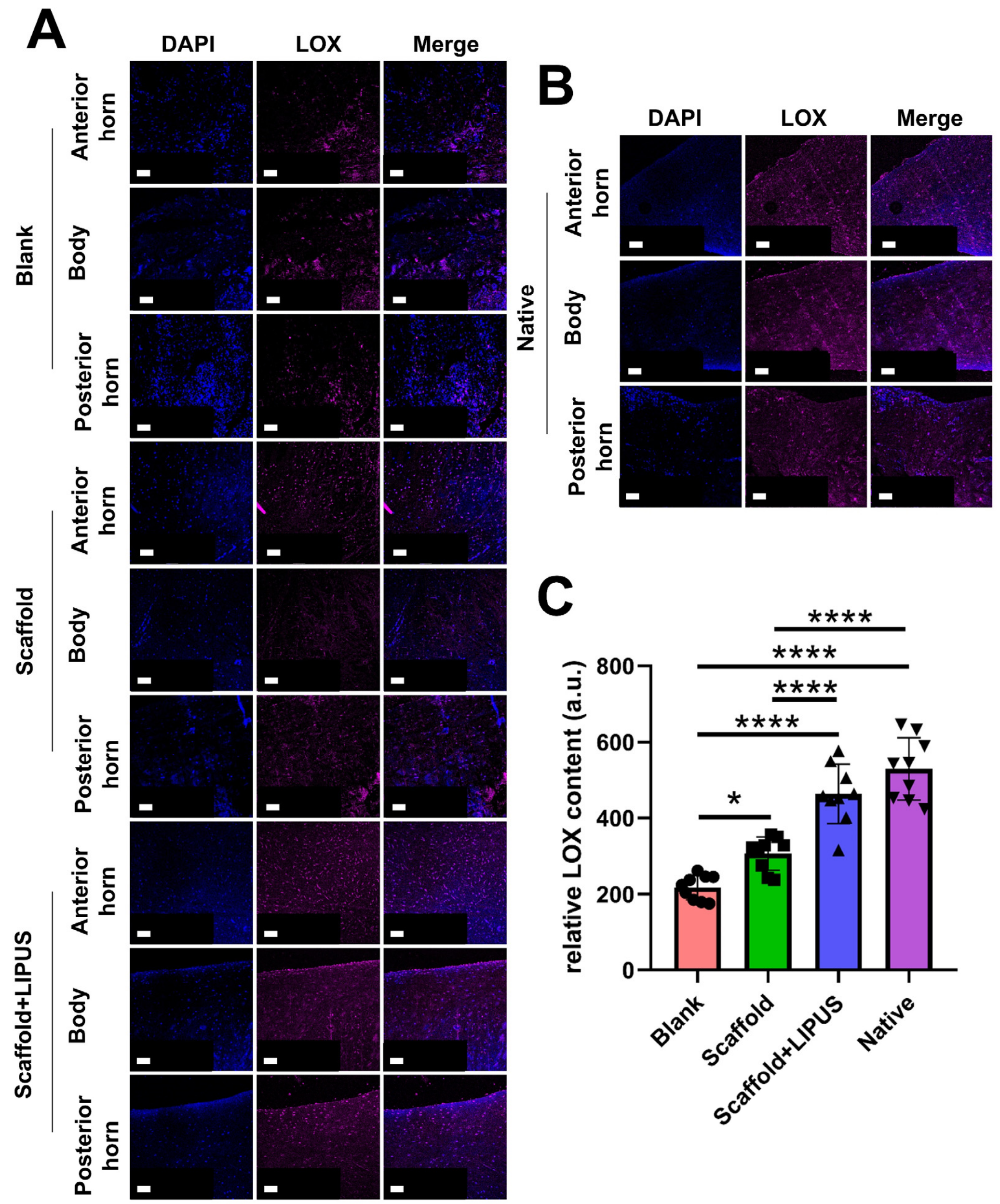

Supplemental Figure 18. The immunofluorescent analysis of LOX in regenerated tissue and native meniscus of beagle canine. (A) the immunofluorescent staining of LOX in regenerated tissue. Scale bar: 50 μm. **(B)** the immunofluorescent staining of LOX in native meniscus. Scale bar: 50 μm. **(C)** the semiquantitative analysis of LOX content, n=9, one-way ANOVA. * represents p<0.05, **** represents p<0.001.

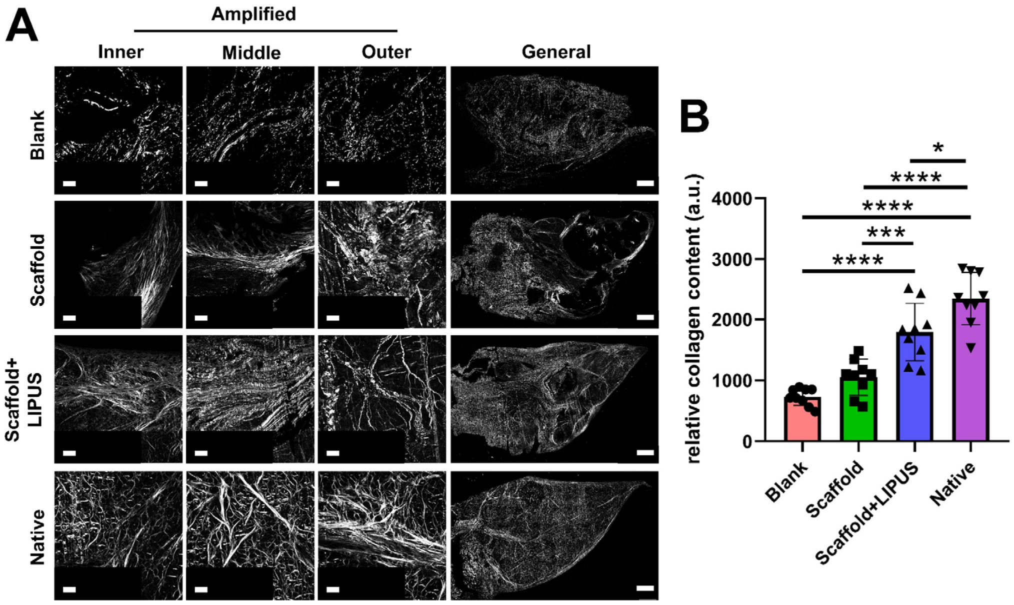

Supplemental Figure 19. The two-photon microscopic analysis of collagen fibers in regenerated tissue and native menisci of beagle canine. (A) the representative second harmonic generation images of collagens. Scale bar of Amplified: 50 µm, Scale bar of General: 500 µm **(B)** the semiquantitative analysis of collagen content reflected by second harmonic generation intensity, n=9, one-way ANOVA. * represents p<0.05, *** represents p<0.005, **** represents p<0.001.

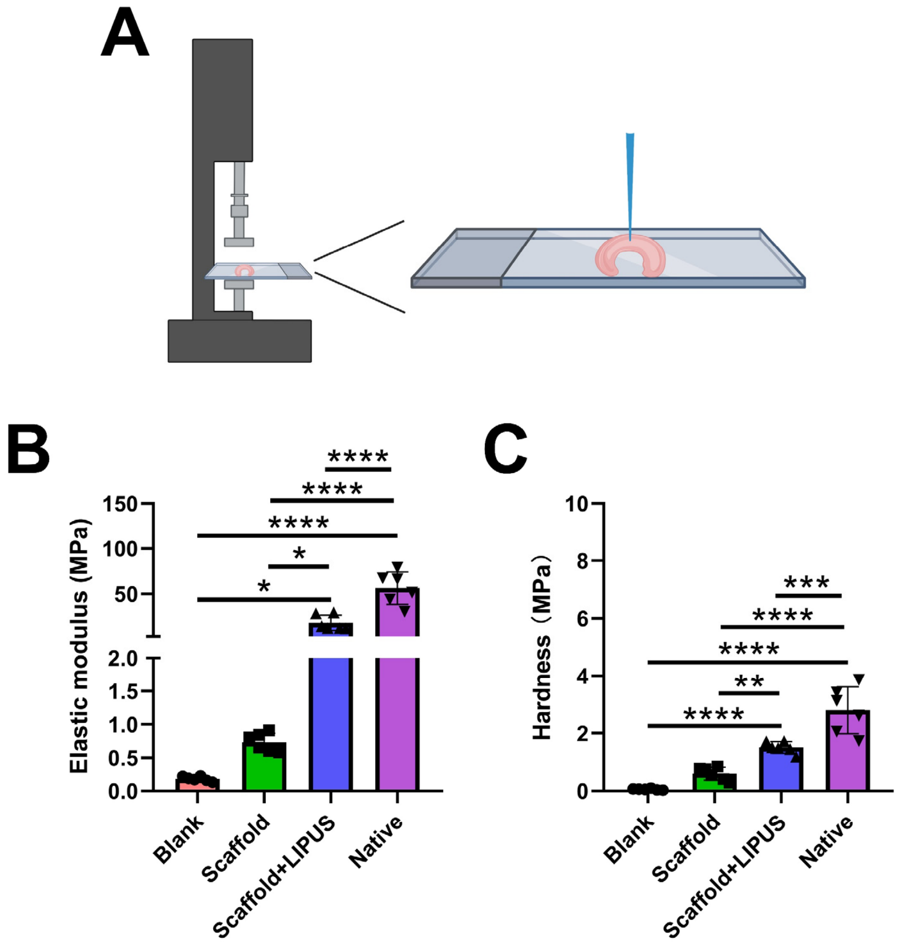

Supplemental Figure 20. The nanoindentation test of regenerated tissue and native menisci of beagle canine. (A) the schematics diagram of nanoindentation test in meniscus tissue. **(B)** the elastic modulus of regenerated tissue and native meniscus. **(C)** the hardness of regenerated tissue and native meniscus, n=6, one-way ANOVA. * represents p<0.05, ** represents p<0.01, *** represents p<0.005, **** represents p<0.001.

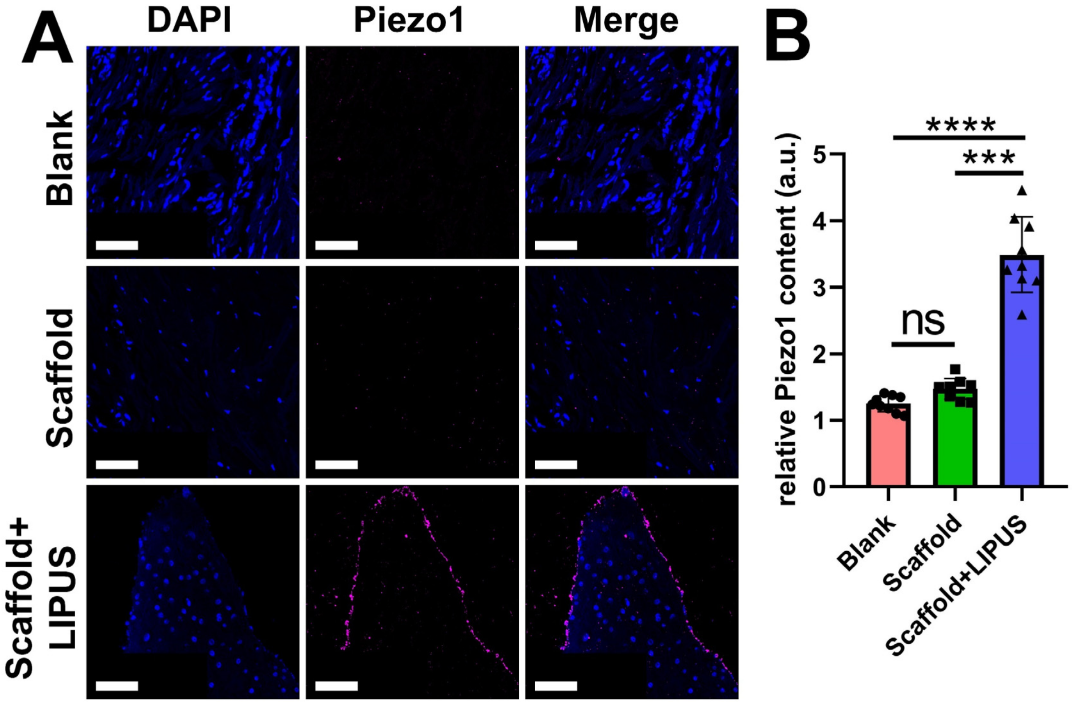

Supplemental Figure 21. The immunofluorescent analysis of Piezo1 within regenerated tissue of beagle canine. (A) the representative immunofluorescent images. Scale bar: 50 μm. **(B)** the semiquantitative analysis of Piezo1, n=9, one-way ANOVA. ns represents no significant difference, *** represents p<0.005, **** represents p<0.001.

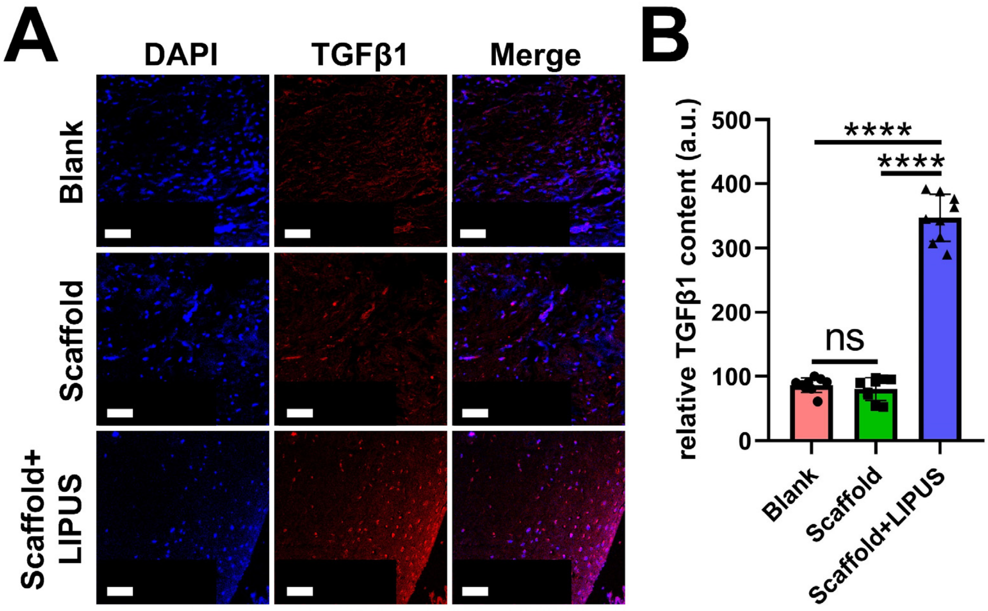

Supplemental Figure 22. The immunofluorescent analysis of TGFβ1 within regenerated tissue of beagle canine. (A) the representative immunofluorescent images. Scale bar: 50 μm. **(B)** the semiquantitative analysis of TGFβ1, n=9, one-way ANOVA. ns represents no significant difference, **** represents *p*<0.001.

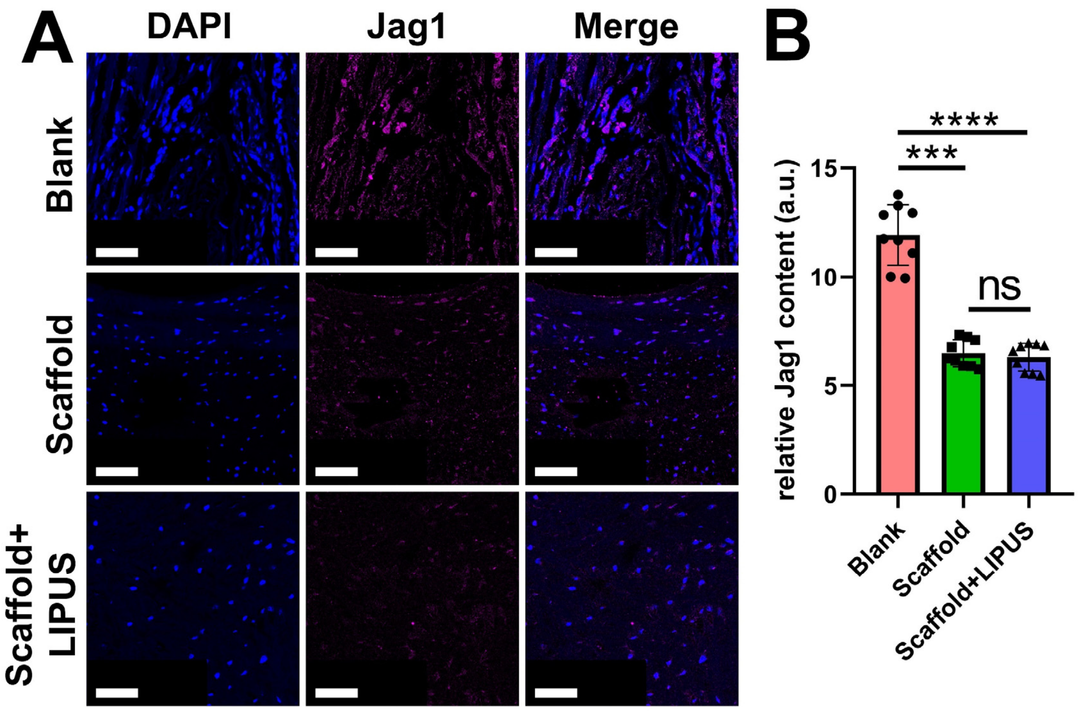

Supplemental Figure 23. The immunofluorescent analysis of Jag1 within regenerated tissue of beagle canine. (A) the representative immunofluorescent images. Scale bar: 50 μm. **(B)** the semiquantitative analysis of Jag1, n=9, one-way ANOVA. ns represents no significant difference, *** represents p<0.005, **** represents p<0.001.

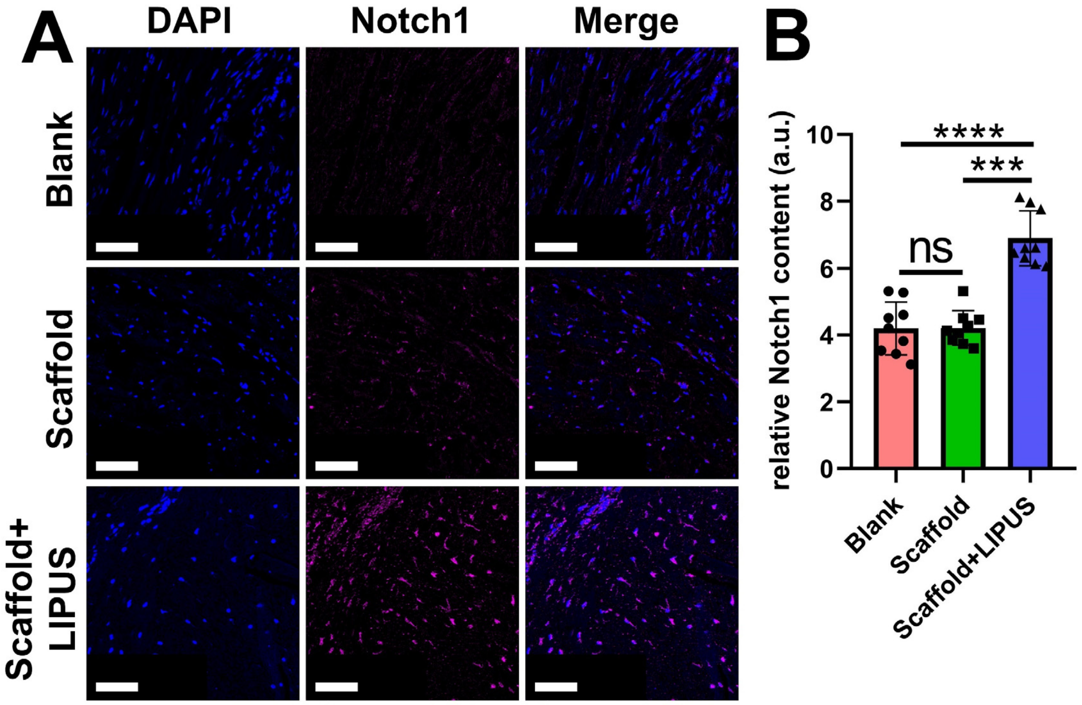

Supplemental Figure 24. The immunofluorescent analysis of Notch1 within regenerated tissue of beagle canine. (A) the representative immunofluorescent images. Scale bar: 50 µm. **(B)** the semiquantitative analysis of Notch1, n=9, one-way ANOVA. ns represents no significant difference, *** represents p<0.005, **** represents p<0.001.

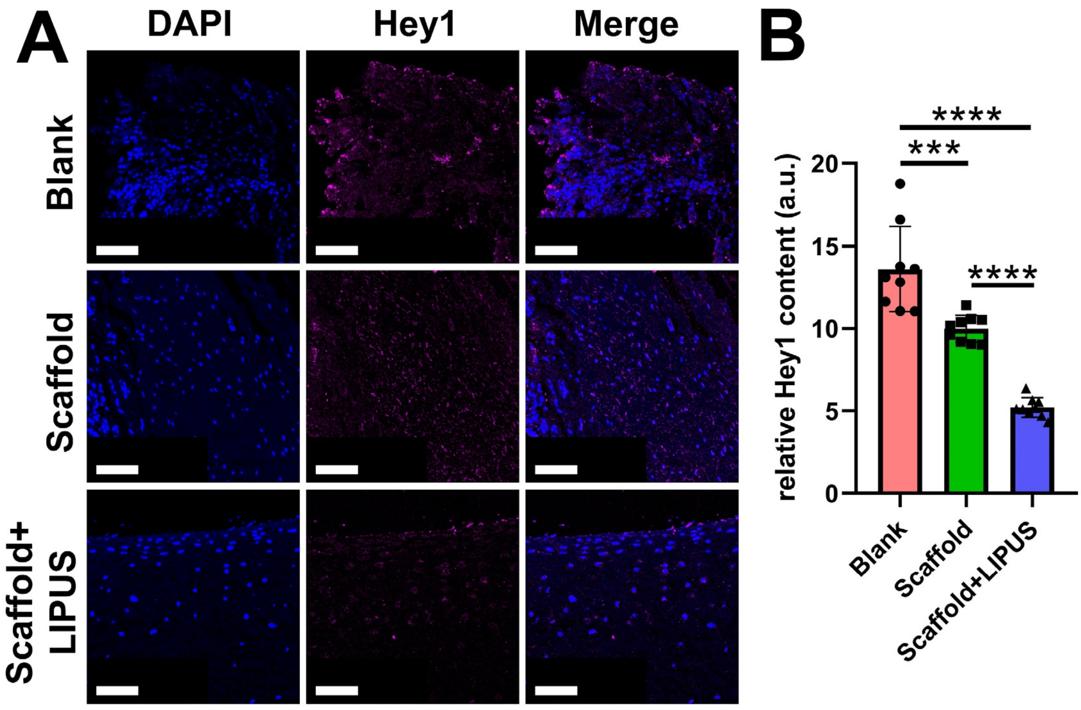

Supplemental Figure 25. The immunofluorescent analysis of Hey1 within regenerated tissue of beagle canine. (A) the representative immunofluorescent images. Scale bar: 50 μm. **(B)** the semiquantitative analysis of Hey1, n=9, one-way ANOVA. *** represents p<0.005, **** represents p<0.001.

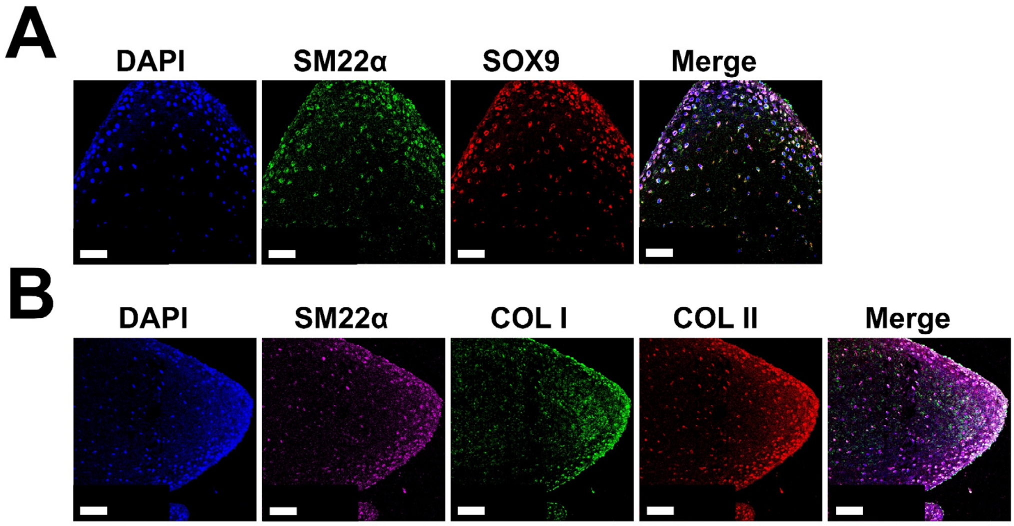

Supplemental Figure 26. The identification of fibrochondrogenic transdifferentiation of smooth muscle cells within regenerated tissue of beagle canine Scaffold+LIPUS group using immunofluorescent colocalization of SM22alpha/SOX9/COL I/COL II. (A) the immunofluorescent co-staining of SM22alpha and SOX9. **(B)** the immunofluorescent co-staining of SM22alpha, COL I and COL II. Scale bar: 50 μm.

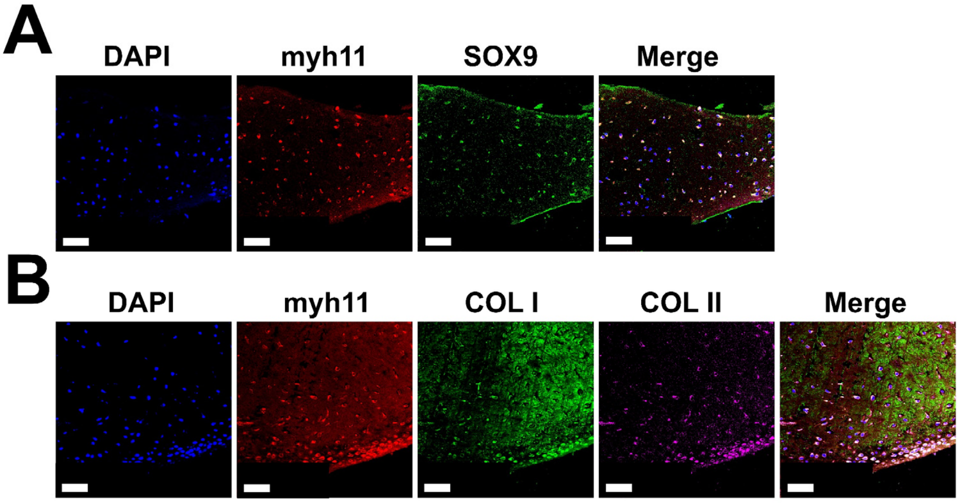

Supplemental Figure 27. The identification of fibrochondrogenic transdifferentiation of smooth muscle cells within regenerated tissue of beagle canine Scaffold+LIPUS group using immunofluorescent colocalization of myh11/SOX9/COL I/COL II. (A) the immunofluorescent co-staining of myh11 and SOX9. **(B)** the immunofluorescent co-staining of myh11, COL I and COL II. Scale bar: 50 μm.

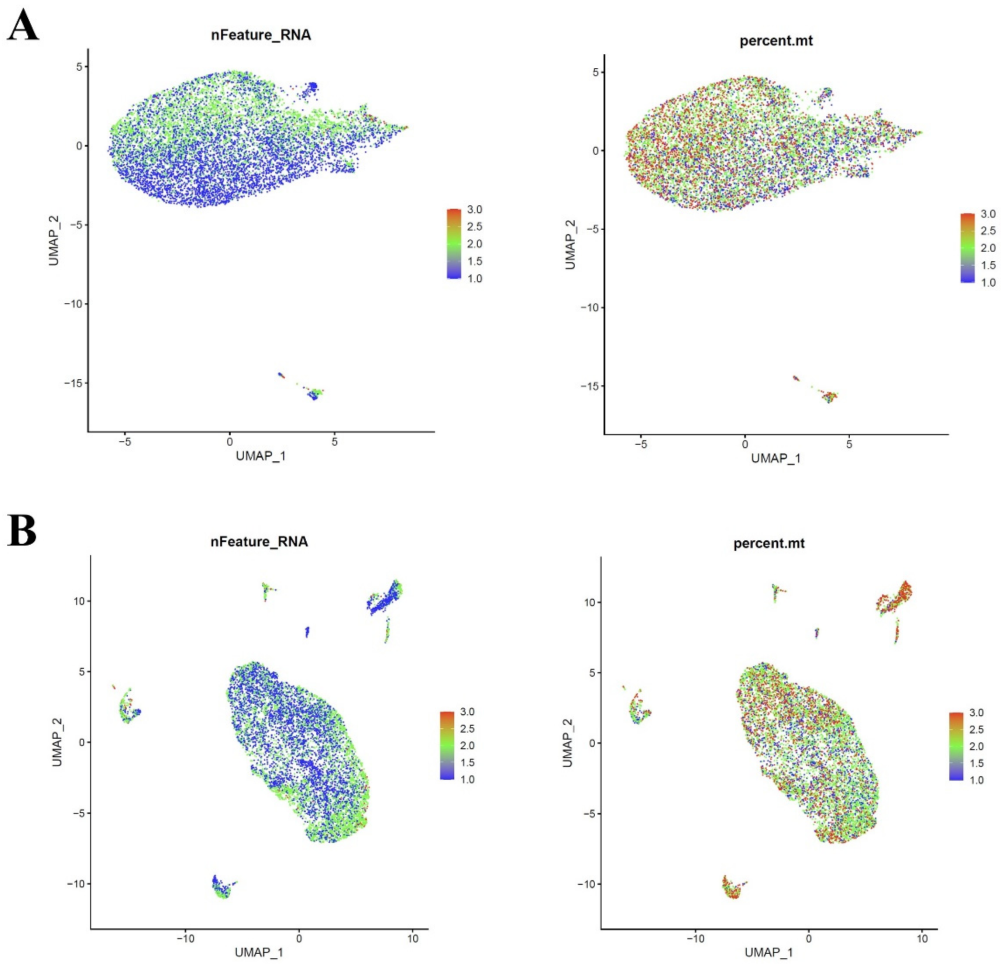

Supplemental Figure 28. The cell quality of beagle canine native and regenerated meniscal tissue. (A) native meniscal tissue. **(B)** regenerated meniscal tissue after LIPUS treatment.

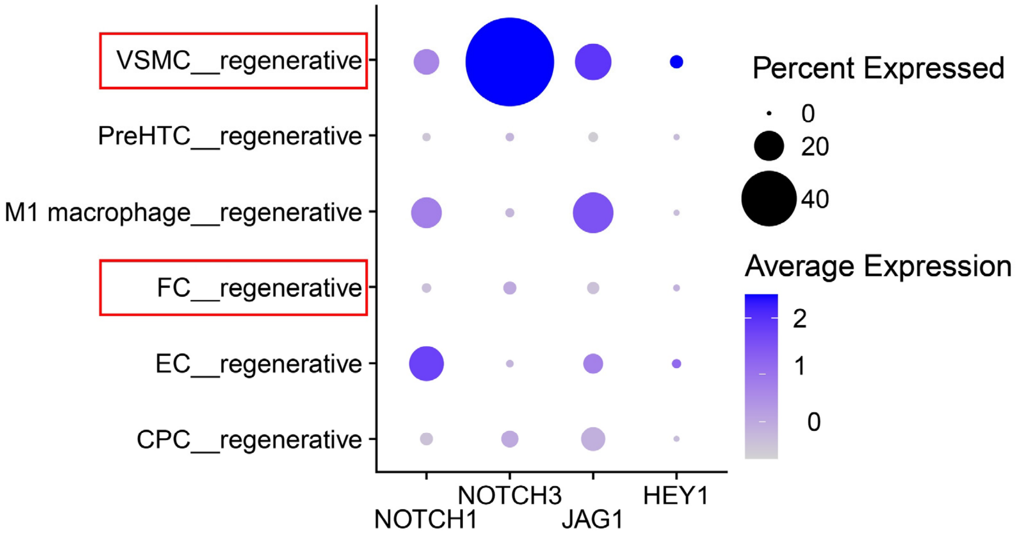

Supplemental Figure 29. The expression patterns of Notch signaling pathway (Notch1, Notch3, Jag1, Hey1) within the context of the scRNA-Seq data of regenerated meniscal tissue of beagle canine after LIPUS treatment.

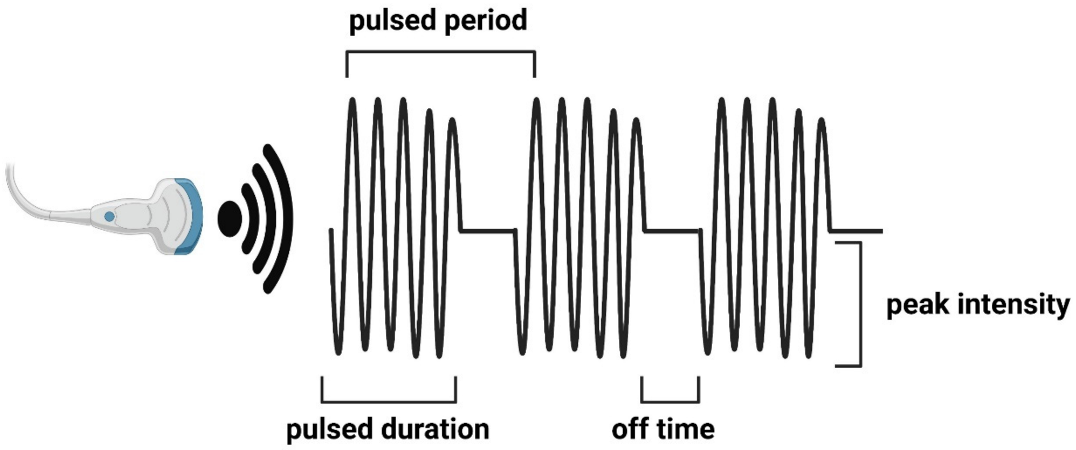

Supplemental Figure 30. The schematic diagram of LIPUS working model.

Supplemental table 1: Abbreviations used

Abbreviations	Definition
VSMCs	vascular smooth muscle cells
TGFβ	transforming growth factor beta
CTGF	connective tissue growth factor
KGN	kartogenin
LIPUS	Low-intensity pulsed ultrasound
FDA	Food and Drug Administration
MSCs	mesenchymal stem cells
scRNA-seq	Single-cell RNA sequencing
SM22α	smooth muscle 22 alpha
α-SMA	Alpha smooth muscle actin
COL II	type II collagen
SOX9	Sry related HMG box-9
Myh11	myosin heavy chain 11
COL I	type I collagen
Gel-MA	methacrylated gelatin
RFP	red fluorescence protein
ISH	in situ hybridization
SRY	sex-determining region Y-linked
ECM	extracellular matrix
GAG	glycosaminoglycans
RUNX1	Runt-related transcription factor 1
KEGG	Kyoto encyclopedia of genes and genomes
Jag1	jagged canonical Notch ligand 1
Dll1	delta like canonical Notch ligand 1
Hey1	hes-related family bHLH transcription factor with YRPW motif 1
PU	polyurethane
PCL	poly-ε-caprolactone

3D	three-dimensional
2D	two-dimensional
OARSI	osteoarthritis cartilage histopathology assessment system
COL VI	type 6 collagen
LOX	lysyl oxidase
SHG	second harmonic generation
MGP	matrix Gla protein
MAPK	mitogen-activated protein kinase
ERK	extracellular signal-regulated kinase
HCS	human chondrocytic cell line
IL-1β	interleukin-1β
mTOR	mammalian target of rapamycin
FDM	Fused deposition manufacturing
PO	polygon offset
LT	layer thickness
IRB	institutional review board
α-MEM	α-Minimum Essential Medium
FBS	fetal bovine serum
EDTA	EthyleneDiamine Tetraacetic Acid
LAP	Phenyl (2,4,6-trimethylbenzoyl) phosphinic Acid Lithium Salt
TBST	Tris-HCL Buffered Saline with Tween 20
ROI	region of interest
PVDF	polyvinylidene fluoride membrane
ECL	enhanced chemiluminescence
HRP	horseradish peroxidase
qPCR	quantitative real-time polymerase chain reaction
PBST	Phosphate Buffered Saline with Tween 20
DAPI	4',6-diamidino-2-phenylindole dihydrochloride
GAPDH	glyceraldehyde-3-phosphate dehydrogenase

PBS	phosphate buffered saline
SSC	sodium citrate buffer
BSA	bovine serum albumin
FITC	Fluorescein isothiocyanate isomer
SD	standard deviation
ANOVA	analysis of variance
MFC	medial femoral condyle
MTP	medial tibial plateau
HE	hematoxylin-eosin
ns	no significant difference
cDNA	complementary DNA
GO	gene ontology
a.u.	arbitrary unit
FC	fibrochondrocytes
RegC	regulatory chondrocytes
TAGLN	transgelin
ACTA2	actin alpha 2, smooth muscle
ACTG2	actin gamma 2, smooth muscle
EC	endothelial cells
PreHTC	prehypertrophic chondrocytes
CPC	cartilage progenitor cells
ACAN	Aggrecan
CCN2	CTGF
SOD2	superoxide dismutase 2
OMD	osteomodulin
MT2A	metallothionein 2A
MT1E	metallothionein 1E
MMP	Matrix Metalloproteinase

Supplemental table 2: Scoring System for Evaluation of the Quality of Meniscal Repair Tissue[a]

	0	1	2	3
Defect filling	No fill	<25%	25–75%	>75%
Surface	No surface	ruptured	Fissured/fibrillated	Meniscus-like
Integration	No integration	Partial, unilateral integration	Bilateral partial or unilateral complete integration	Bilateral complete integration
Cellularity	No cells	>10 cell cluster/slide	No cell cluster/slide, Cell-ECM-ratio> 0.5	Meniscus-like cell-ECM ratio
Cell morphology	No cells	<25% meniscus-like cells	25%-75% meniscus-like cells	>75% meniscus-like cells
Content of proteoglycan	No staining for proteoglycan	<25%	25–75%	>75%
Content of type 2 collagen	No staining for type 2 collagen	<25%	25–75%	>75%
Stability	No stability	weak	Stable in shape	Stable to pressure and pulling stress

[a]ECM, extracellular matrix.

Supplemental table 3. The information and arrangement of beagle canine

Number	Knee	Group	Application
#1	Left	meniscectomy (blank) group	Histological analyses of regenerated meniscal tissue and cartilage
	Right	meniscectomy (blank) group	Histological analyses of regenerated meniscal tissue and cartilage
#2	Left	meniscectomy (blank) group	Histological analyses of regenerated meniscal tissue and cartilage
	Right	meniscectomy (blank) group	Histological analyses of regenerated meniscal tissue and cartilage
#3	Left	meniscectomy (blank) group	Histological analyses of regenerated meniscal tissue and cartilage
	Right	meniscectomy (blank) group	Histological analyses of regenerated meniscal tissue and cartilage
#4	Left	meniscectomy (blank) group	Nanoindentation of regenerated meniscal tissue
	Right	meniscectomy (blank) group	Nanoindentation of regenerated meniscal tissue
#5	Left	PU_PCL scaffold group	Histological analyses of regenerated meniscal tissue and cartilage
	Right	PU_PCL scaffold group	Histological analyses of regenerated meniscal tissue and cartilage
#6	Left	PU_PCL scaffold group	Histological analyses of regenerated meniscal tissue and cartilage
	Right	PU_PCL scaffold group	Histological analyses of regenerated meniscal tissue and cartilage
#7	Left	PU_PCL scaffold group	Histological analyses of regenerated meniscal tissue and cartilage
	Right	PU_PCL scaffold group	Histological analyses of regenerated meniscal tissue and cartilage
#8	Left	PU_PCL scaffold group	Nanoindentation of regenerated meniscal tissue
	Right	PU_PCL scaffold group	Nanoindentation of regenerated meniscal tissue
#9	Left	PU_PCL scaffold+LIPUS group	Histological analyses of regenerated meniscal tissue and cartilage
	Right	PU_PCL scaffold+LIPUS group	Histological analyses of regenerated meniscal tissue and cartilage
#10	Left	PU_PCL scaffold+LIPUS group	Histological analyses of regenerated meniscal tissue and cartilage
	Right	PU_PCL scaffold+LIPUS group	Histological analyses of regenerated meniscal tissue and cartilage
#11	Left	PU_PCL scaffold+LIPUS group	Nanoindentation of regenerated meniscal tissue
	Right	PU_PCL scaffold+LIPUS group	Nanoindentation of regenerated meniscal tissue
#12	Left	PU_PCL scaffold+LIPUS group	Single cell RNA-seq of regenerated meniscal tissue
	Right	PU_PCL scaffold+LIPUS group	Single cell RNA-seq of regenerated meniscal tissue
#13	Left	sham group	Histological analyses of regenerated meniscal tissue and cartilage
	Right	sham group	Histological analyses of regenerated meniscal tissue and cartilage
#14	Left	sham group	Histological analyses of regenerated meniscal tissue and cartilage
	Right	sham group	Histological analyses of regenerated meniscal tissue and cartilage
#15	Left	sham group	Nanoindentation of regenerated meniscal tissue
	Right	sham group	Nanoindentation of regenerated meniscal tissue
#16	Left	sham group	Single cell RNA-seq of native meniscal tissue
	Right	sham group	Single cell RNA-seq of native meniscal tissue

Supplemental table 4: Key resources table

Reagent or resource	Source	Identifier
**Primary antibodies**		
Goat anti α-SMA antibody	Abcam	ab21027
Rabbit anti SM22α antibody	Abcam	Ab14106
Mouse anti SOX9 antibody	Abcam	Ab238053
Rabbit anti SOX9 antibody	Sigma	HPA001758
Mouse anti collagen II monoclonal antibody	Invitrogen	MA5-13026
Mouse anti Myh11 antibody	MilliporeSigma	M7786
Rabbit anti collagen II antibody	Abcam	ab34712
Goat anti collagen I antibody	Arigo	ARG21965
Rat anti RFP antibody	Chromotek	5F8
FITC-labeled anti-biotin	MilliporeSigma	F6762
Mouse anti aggrecan antibody	MilliporeSigma	C8035
Rabbit anti RUNX1 antibody	Proteintech	25315-1-AP
Mouse anti GAPDH antibody	Proteintech	60004-1-Ig
Rabbit anti Piezo1 antibody	Proteintech	28511-1-AP
Rabbit anti Jag1 antibody	CST	D4Y1R
Rabbit anti Notch1 antibody	CST	D6F11
Rabbit anti Hey1 antibody	Proteintech	19929-1-AP
Mouse anti TGF beta1 antibody	Abcam	ab190503
Moue anti Smad2/3 antibody	SANTA CRUZ	Sc-133098
Rabbit anti phospho-Smad2 (Ser465/467)/Smad3 (Ser423/425) antibody	CST	8828
Rat anti perlecan antibody	Abcam	ab2501
Rabbit anti collagen VI antibody	Abcam	ab6588
Rabbit anti LOX polyclonal antibody	Proteintech	17958-1-AP
**Secondary antibodies**		
Donkey anti-rabbit IgG H&L (Alexa Fluor® 647)	Abcam	ab150075
Donkey anti-mouse IgG H&L (Alexa Fluor® 594)	Abcam	ab150108
Donkey anti-goat IgG H&L (Alexa Fluor® 488)	Abcam	ab150129
Donkey anti-rat IgG H&L (Alexa Fluor® 488)	Abcam	ab150153
Goat anti-mouse IgG H&L (HRP)	Abcam	ab6789
Goat anti-rabbit IgG H&L (HRP)	Abcam	ab6721
Donkey anti-Goat IgG H&L (HRP)	Abcam	ab6885
**Chemicals and Recombinant proteins**		
YODA1	MCE	HY-18723
GsMTx-4	MCE	HY-P1410
Gel-MA	aladdin	M299511
LAP	SunP Biotech	SP-BI-C02
Recombinant human TGFβ1	Peprotech	100-21
Type I collagenase	Gibco	17100017
Type II collagenase	Gibco	17101015
**Transgenic mice model**		

B6.FVB-*Tg(Myh11-creERT2)Smoc*	Shanghai Model Organisms Center, Inc.	NMX-TG-192009
C57BL/6JSmoc-*Gt(ROSA)26Sor*$^{em(CAG\text{-}LSL\text{-}tdTomato)1Smoc}$	Shanghai Model Organisms Center, Inc.	NM-KI-225042
**Software**		
GraphPad Prism software	GraphPad	version 8.0.1
Image J software	NIH	N/A
Mimics software	Materialise	version 19.0

Supplemental table 5: the summary of gene expression in scRNA-Seq

Sample	Estimated Number of Cells	Fraction Reads in Cells	Mean Reads per Cell	Median Genes per Cell	Total Genes Detected	Median UMI Counts per Cell
native	11523	75.47%	31,888	1832	32968	5273
regenerative	10891	78.98%	36,660	2608	31872	7968

Supplemental table 6: Primer sequences for rat VSMCs

Gene	Forward (5′-3′)	Reverse (5′-3′)
GAPDH	AAGTTCAACGGCACAGTCAAGG	GACATACTCAGCACCAGCATCAC
COL1A1	GTGCGATGGCGTGCTATGC	CTATGACTTCTGCGTCTGGTGATAC
COL2A1	ACGCTCAAGTCGCTGAACAAC	AATCCAGTAGTCTCCGCTCTTCC
Aggrecan	GAACCTTCGCTCCAATGACTCTG	GGCTTCGCTGTCCTCAATGC
SOX9	GGCGGAGGAAGTCGGTGAAG	AGATGGCGTTAGGAGAGATGTGAG
RUNX1	AGACCCTGCCCATCGCTTTC	TTGAATCTCGCCACTTGGTTCTTC
COL10A1	GGATGCCTCTTGTCAGTGCTAAC	GTCATAGTGCTGCTGCCTGTTG
Myocardin	CTGAAGAGCCCGCAACACATC	CCAGAGGAGGAAGCCAGGAAG
Myh11	AGCAGGAAGAGTACCAACGAGAG	GCCGCTCAATCAGTTCAATACAAG
SM22alpha	GGCTAGTGGAGTGGATTGTAATGC	TGTTCACCAACTTGCTCAGAATCAC
Alpha-SMA	CCACTGCTGCTTCCTCTTCTTC	TGCCCGCCGACTCCATTC
Piezo1	CGGACAGTGAGGAGGAAGAGGAG	CCTGTTCACGACGCTGCCTTAG
Jag1	TGGCTTGGGTCTGTTGCTTGG	CATTGTTGGTGGTGTTGTCCTCAG
Notch1	AGACAGGCAACAGTGAGGAAGAAG	TGGCAGCATCTGAACGAGAGTATC
Notch2	GAAGGTGGCACGCAGGTCTC	AGCACTGGTCTGAGTCTTGAACAC
Notch3	GTTCCCAGCGAGCATCCTTATTTG	GGCGTTGAGTCGGACCAGTC
Notch4	CCTGCTGGTGGTGCTGAGTC	TGTTCCTGCCTTCACTGTCCTTC
Hey1	GGCTATGGACTATCGGAGTTTGGG	AGGCGAACACGAAGCGGATC
Hey2	CGGGAGGCAGCAGTGATGAC	ATAGGCGACATGGCGTTGACTC
Hes1	CGGCCAATTTGCTTTCCTCATCC	TGGAAGGCGACACTGCGTTAG

TGFbeta1	TAGCAACAATTCCTGGCGTTACC	CCTGTATTCCGTCTCCTTGGTTC
Smad2	GTCGTCCATCTTGCCATTCACTC	GTTCTCCACCACCTGCTCCTC
Smad3	AGGGCTTTGAGGCTGTCTACC	TGCTGGTCACTGTCTGTCTCC
